# Drug Repurposing for COVID-19 using Graph Neural Network with Genetic, Mechanistic, and Epidemiological Validation

**DOI:** 10.21203/rs.3.rs-114758/v1

**Published:** 2020-12-11

**Authors:** Kanglin Hsieh, Yinyin Wang, Luyao Chen, Zhongming Zhao, Sean Savitz, Xiaoqian Jiang, Jing Tang, Yejin Kim

**Affiliations:** 1 Center for Secure Artificial Intelligence for Healthcare, School of Biomedical Informatics, The University of Texas Health Science Center at Houston, Houston, TX, USA; 2 Research Program in Systems Oncology, Faculty of Medicine, University of Helsinki, Helsinki, Finland; 3 Center for Precision Health, School of Biomedical Informatics, The University of Texas Health Science Center at Houston, Houston, TX, USA; 4 Institute for Stroke and Cerebrovascular Disease, The University of Texas Health Science Center at Houston, Houston, TX, USA

## Abstract

Amid the pandemic of 2019 novel coronavirus disease (COVID-19) infected by SARS-CoV-2, a vast amount of drug research for prevention and treatment has been quickly conducted, but these efforts have been unsuccessful thus far. Our objective is to prioritize repurposable drugs using a drug repurposing pipeline that systematically integrates multiple SARS-CoV-2 and drug interactions, deep graph neural networks, and in-vitro/population-based validations. We first collected all the available drugs (n= 3,635) involved in COVID-19 patient treatment through CTDbase. We built a SARS-CoV-2 knowledge graph based on the interactions among virus baits, host genes, pathways, drugs, and phenotypes. A deep graph neural network approach was used to derive the candidate drug’s representation based on the biological interactions. We prioritized the candidate drugs using clinical trial history, and then validated them with their genetic profiles, in vitro experimental efficacy, and electronic health records. We highlight the top 22 drugs including Azithromycin, Atorvastatin, Aspirin, Acetaminophen, and Albuterol. We further pinpointed drug combinations that may synergistically target COVID-19. In summary, we demonstrated that the integration of extensive interactions, deep neural networks, and rigorous validation can facilitate the rapid identification of candidate drugs for COVID-19 treatment.

## One Sentence Summary

Deep learning and comprehensive evaluation identifies Azithromycin and Atorvastatin as repurposable drugs for COVID-19.

## INTRODUCTION

The emergence of SARS-CoV-2 (2019 novel coronavirus, COVID-19) has created a global pandemic. As of today (August 27, 2020), there have been over 24 million COVID-19 cases worldwide, but no vaccine or highly effective antiviral treatment for COVID-19 patients is available yet (1). While many millions more will likely be infected, some pessimistic estimation is that it may take at least one year for an approved effective vaccine to be in place (2). Lack of vaccine or antiviral drugs with clinical efficacy substantiates the need to expand research efforts in the prevention and/or treatment for COVID-19 (3, 4). There has been great effort in this direction and researchers have screened thousands of candidate agents (5, 6). These agents can be divided into two broad categories, those that can directly target the virus replication cycle, and those based on immunotherapy approaches either aimed to boost innate antiviral immune responses (e.g., targeting the host angiotensin-converting enzyme 2 (ACE2) that SARS-CoV-2 directly binds (7)) or to alleviate damage induced by dysregulated inflammatory responses (8). Research on this rapidly emerging infectious disease has created valuable knowledge. For example, a curated list of potential COVID-19 therapeutics is available for research, such as Comparative Toxicogenomics Database (CTDbase) or PharmGKB (9), which have offered valuable resources for systematic integration of accumulated knowledge (10).

Drug discovery, however, is an expensive and time-consuming process. It typically takes many years and costs billions of dollars to develop and obtain the approval of a drug. Drug repurposing is to identify existing drugs or compounds that can be efficacious to other conditions of interest. Drug repurposing via systematic integration of pharmacodynamics, in vitro drug screening, and population-scale clinical data analysis carries high potential for a novel approach by identifying highly promising drugs and their combinations to save the cost and accelerate discovery (11). Based on this accumulated genomic and pharmacological knowledge, several computational approaches have explored and identified potentially effective drug and/or vaccine candidates (12). Examples include a network proximity study in protein-protein interaction (PPI) networks (13-15), in silico protein docking (16), and sequencing analysis (17). Another family of studies has utilized retrospective analysis of clinical data, such as electronic health records (EHRs) (2). These studies have assessed the potential efficacy of drugs including angiotensin receptor blockers, estradiol, or antiandrogens (2, 18). Although the network pharmacology and the retrospective clinical data analysis provide complementary insight into potential drugs (19), few studies have investigated by integrating these complementary perspectives, particularly in COVID-19. This work attempts to identify repurposable drugs from SARS-CoV-2-drug interactions and validating the drugs from in vitro efficacy and large-scale clinical data to prioritize repurposable drugs.

In this work, we innovated the traditional network analysis by deep graph neural representation to broaden the scope from local proximity to global topology. In traditional network analysis, network proximity is defined with explicit and direct interactions (13, 14), thus a node’s local role (e.g., neighbors, edge directions) and global position (e.g., overall topology or structure) are less considered. With the recent advancement in machine learning and representation learning, the graph neural network (GNN) approach is mature for the application of its state-of-the-art technology to network pharmacology. GNN is one field of deep neural networks that derive a vectorized representation of nodes, edges, or whole graphs. The graph node embedding can preserve the node’s local role and global position in the graph via iterative and nonlinear message passing and aggregation. It learns the structural properties of the neighborhood and the graph’s overall topological structure (20). Adopting GNN into the biomedical network facilitates the integration of multimodal and complex relationships. Recently GNN has shown a great promise in predicting interactions (e.g., PPIs, drug-drug adverse interactions, and drug-target interactions) and discovery of new molecules (21, 22). GNN can also benefit drug repurposing by representing the complex interaction between drugs and diseases. A recent attempt has been made to use the GNN for drug repurposing, which builds a general biomedical knowledge graph, called Drug Repurposing Knowledge Graph (DRKG), from seven biomedical databases and utilizes the embedding to discover a therapeutic association between drugs and diseases (15). The knowledge graph includes 15 million edges across 39 different types connecting drugs, disease, genes, and pathways from seven databases including DrugBank, Hetionet, STRING, and a text-mining-driven database (23). This biomedical network representation offers a general and universal understanding of the interaction between drugs, genes, and diseases.

**Figure 1.**
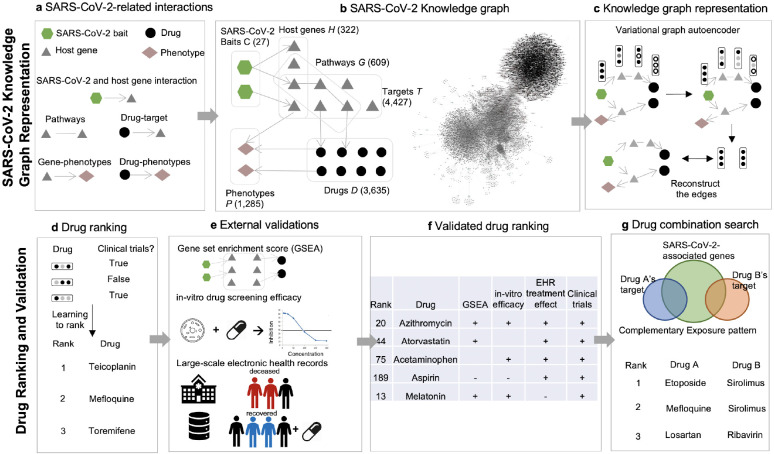
Study workflow. (a) We collected 27 SARS-CoV-2 baits, 322 host genes interacting with baits, 1,783 host genes on 609 pathways, 3,635 drugs, 4,427 drugs’ targets, and 1,285 phenotypes, and their corresponding interactions from a curated list of COVID-19 literature in CTDbase (24). (b) We built the SARS-CoV-2 knowledge graph with nodes (baits, host genes, drugs, targets, pathways, and phenotypes) and edges (virus-host protein-protein interaction, gene-gene in pathways, drug-target, gene-phenotype, drug-phenotype interaction). (c) We derived the node’s embedding using the multi-relational and variational graph autoencoder (25, 26). We transferred extensive representation in DRKG using transfer learning. (d) We built a drug ranking model based on the drug’s embedding as features and clinical trials as silver-standard labels. (e) The drug ranking was validated using drug’s gene profiles , in vitro drug screening efficacy (6), and large-scale electronic health records. (f) We presented validated drugs with their genetic, mechanistic, and epidemiological evidence. (g) Using the highly ranked drug candidates, we searched for drug combinations that satisfy complementary exposure patterns (13).

In this study, we built the SARS-CoV-2 Knowledge Graph from curated COVID-19 literature, transferred the universal representation from DRKG, and then utilized deep GNN to derive repurposable drugs’ representations which were rigorously validated with in vitro efficacy and large-scale EHRs (Fig. 1). Compared to the existing studies (13, 15), our work’s novelty can be summarized as: i) the knowledge graph was tailored to COVID-19 literature as well as backed by universal biomedical literature, ii) the data were comprehensive and up-to-date knowledge on COVID-19 (baits-genes-drugs interactomes, gene expression, in-vitro efficacy, EHRs, and clinical trials), and ii) synergistic drug combinations were identified based on complementary drug targets.

## RESULTS

### Building the SARS-CoV-2 knowledge graph

We built a comprehensive graph, named as the SARS-CoV-2 Knowledge Graph, that represents interactions between SARS-CoV-2 baits, host genes, pathways, targets, drugs (including compounds), and phenotypes (Fig. 1b). We identified drug-target interactions, pathways, gene/drug-phenotype interactions from CTDbase (24). We collected the SARS-CoV-2 and host PPIs from a recent systematic PPI experimental study for SARS-CoV-2 (Methods) (27). The graph had four types of nodes and five types of edges based on the interactions. The four types of nodes include 27 virus baits, 5,677 unique host genes (from 322 host preys, 1,783 genes on pathways, and 4,427 drug targets, Fig. S1a), 3,635 drugs, and 1,285 phenotypes. The five types of edges include 330 virus-host PPIs, 13,423 pairwise genes on the same pathway, 16,972 drug-target pairs, 1,401 gene-phenotype pairs, and 935 drug-phenotype pairs.

### Drug embedding using graph neural network

Using the SARS-CoV-2- knowledge graph, we derived embedding for each drug, gene, phenotype, and SARS-CoV-2 bait. The graph embedding method was the variational graph autoencoder with multi-relational edges (Methods) (26). We set the embedding size as 128 after several trials. We further boosted the representativeness of the embedding by transferring DRKG universal embedding to our embedding. The DRKG embedding contains general biological knowledge (e.g., drug embedding was derived from molecular structures, targets, anatomical therapeutic chemical classifications, side effects, pharmacologic classes, and treating diseases) (15). By transferring the rich representation of DRKG to the SARS-CoV-2 knowledge graph, we can derive embeddings that are more faithful to underlying pharmacokinetics and pharmacodynamics. To this end, we initialized the SARS-CoV-2 knowledge graph node embedding with DRKG embedding and fine-tuned the node embedding by updating them via GNN’s message passing and aggregation (Methods, Note S1).

We first internally validated the confidence of our knowledge graph embedding via link prediction to confirm if the node embedding can capture the network topology centered by SARS-CoV-2. We measured an accuracy to predict interactions between the nodes (SARS-CoV-2 baits, genes, drugs, and phenotypes). We randomly selected 10% of the edges for validation. As a result, our node embedding showed high accuracy in predicting the interactions in the SARS-CoV-2 knowledge graph. The initial DRKG universal embedding (without fine-tuning) achieved 0.5695 AUROC and 0.6431 AUPRC. After fine-tuning the DRKG embedding to the SARS-CoV-2 knowledge graph, we achieved AUROC 0.8121 and AUPRC 0.8524, respectively (Table S1).

**Figure 2.**
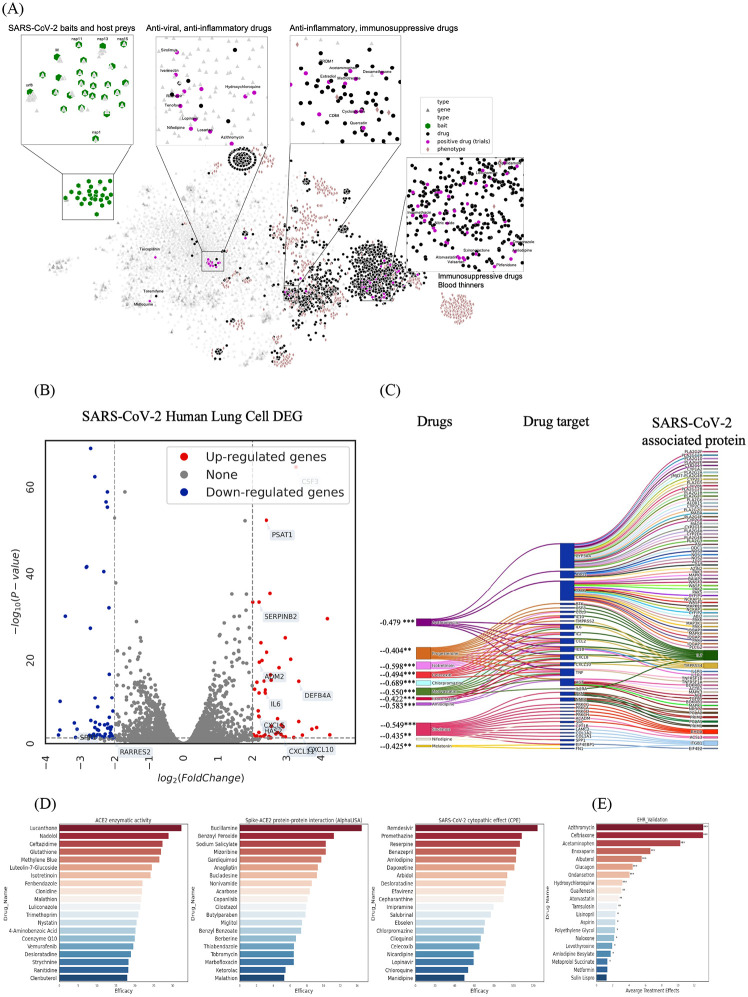
(a) SARS-CoV-2 knowledge graph t-SNE plot. Two nodes that have similar embedding are closely located in the t-SNE plot. We highlighted drugs undergoing clinical trials (as of July 23, 2020) to glimpse the promising repurposable drugs around the trial drugs. SARS-Cov-2 baits were the upper-left green hexagons (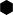). Genes, the gray triangles (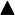), were in the middle between baits and drugs. Drugs, the black rounds (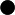), were mixed with genes. Drugs undergoing clinical trials, the purple rounds, were closely located together. Phenotypes, the light brown diamonds (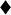), are closely located relevant genes and drugs. We validated the drug ranking using four different external validation sources including (b) Differentially expressed genes in SARS-CoV-2-infected human lung cells (GSE153970). Potential drugs can treat COVID-19 by inhibiting up-regulated genes or activating down-regulated genes. (c) GSEA score between the infected human lung cell transcriptome and drug-induced transcriptome. (d) in-vitro efficacy (e.g. % inhibition in viral entry and cytopathic effect assays (6)), and (e) treatment effect in EHRs (Optum^®^ de-identified EHR database (2007-2020)).

We visualized the 128-dimensional node embedding using t-Distributed Stochastic Neighbor Embedding (t-SNE) to observe the node embedding better (28). The t-SNE plot projects a high-dimensional vector into a low-dimension vector while preserving the pairwise similarity between nodes, thus allowing us to examine the high-dimensional node embedding with low-dimension (e.g., 2-dimensions) visualization. In the t-SNE plot (Fig. 2a, Fig. S2), we found that the node embedding of SARS-CoV-2 baits, host genes, drugs, and phenotypes were distributed separately. We found that a group of antiviral and anti-inflammatory drugs (including Tenofovir, Ritonavir) was closely located to SARS-CoV-2 baits. Another group of anti-inflammatory and immunosuppressive drugs was highlighted including Cyclosporine and Dexamethasone, which were surrounded by genes related to inflammation and infection such as *CD68* and *PRDM1*. We also found a group of blood thinners (Heparin), anti-hypertensives (Amlodipine), anti-platelet (Dipyridamole), and anti-inflammatory drugs (Indomethacin).

### Initial drug ranking

Using the rich representation of the candidate drugs, we built an initial ranking model that predicts antiviral effectiveness. We hypothesized that, because drugs testing in clinical trials are potentially efficacious in treating COVID-19, a drug that is similar to these trial drugs can have potential efficacy too. This drug ranking was an initial filtering step to select possibly potent drugs out of 3,635 candidates. For the labels, 99 clinical trial drugs were matched to the 3,635 drugs. The remaining drugs without matched clinical trials were regarded as having negative efficacy. We designed a customized neural network ranking model based on Bayesian pairwise ranking loss (Methods) (29). The ranking model accuracy was AUROC between 0.77-0.90 and AUPRC between 0.17-0.25 (Table 1). The SARS-CoV-2 knowledge graph embedding that was boosted by general embedding from DRKG showed the highest accuracy, thanks to rich representation in DRKG.

### Multiple-source validation

From the initial drug ranking, we selected the top 300 highly-ranked drugs as potential repurposable candidates. We validated the highly-ranked drugs using a wide spectrum of validation sources such as genetic, mechanistic, and epidemiological evidence, which reflects complementary aspects of drug effectiveness. Note that we did not exclude the clinical trial drugs that were used in training.

### Genetic validation using gene set enrichment analysis

For the genetic validation, we compared the gene expression signature profiles of candidate drugs with that of SARS-CoV-2-infected host cells. We used gene set enrichment analysis (GSEA) to identify a significant association between SARS-CoV-2 and candidate drugs (30). We obtained the gene expression signature of SARS-CoV-2 from SARS-CoV-2 infected human lung cells (Fig. 2.B) (31), and obtained the drug's gene expression signature profile from the Connectivity Map (cMAP) database (GSE92742 and GSE70138) (32). We determined whether the drug’s gene expression signature is negatively correlated with that of SARS-CoV-2 based on the enrichment score (ES) (33). The combining ES <0 and *p*-value <0.05 was considered as the threshold to determine that a drug may inhibit the up-regulated or activate the down-regulated host genes (Methods, Note S4). As a result (Fig. 2.C), we identified 183 statistically significant drugs including Azithromycin (ES=−0.479), Progesterone(ES=−0.404), Amodipine (ES=−0.583), Atorvastatin (ES=−0.550), and Nifedipine (ES=−0.435).

### Retrospective in-vitro drug screening validation

We validated the candidates by comparing them with in vitro drug screening results retrospectively. We collected four different drug screening studies that target viral entry (ACE2 enzymatic activity, Spike-ACE2 protein-protein interaction and viral replication/infection (Fig. 2D) (cytopathic effect, two different compound libraries) (5, 6). Details on identifying efficacious drugs in each cell assay are described in Methods. We calculated precision and recall between the predicted (top 300 highly-ranked) drugs and the efficacious drugs in each screening result. We focused on only those drug candidates that are included in the compound library in the screening study. As a result, the recall was between 0.21 and 0.44 and the precision was between 0.04 and 0.18 (Table 2). Caution is needed in interpreting the accuracy here, because the number of overlapping drugs is limited in some studies and, thus, the statistical power is limited.

### Population-based validation

We examined drugs in EHRs of COVID-19 patients. We used the Optum^®^ de-identified EHR database (2007-2020). We derived efficacious drugs from EHRs that reduce the risk of mortality among hospitalized COVID-19 patients. We calculated drugs’ averaged treatment effect among treated (ATT) with inverse propensity score matching (PSM) and weighting from 34,043 COVID-19 recovered or deceased patients (Table 3, Fig. S3b, see Methods). As a result, EHRs had a total of 391 drugs used for COVID-19 hospitalized patients; 138 drugs were common in EHRs and our initial 3,635 drugs. Ten (out of 138) drugs were effective (ATT>0 and *p*-value<0.05) in the EHRs (Fig. 2E). Among the ten positive drugs, our method identified several positive drugs with statistical significance: Azithromycin (ATT=13.08), Ceftriaxone (ATT=13.05), Acetaminophen (ATT=10.27), Albuterol (ATT=5.55), Glucagon (ATT=4.42), and Hydroxychloroquine (ATT=3.18) (Table S2).

### Validated high-ranked drugs

**Figure 3.**
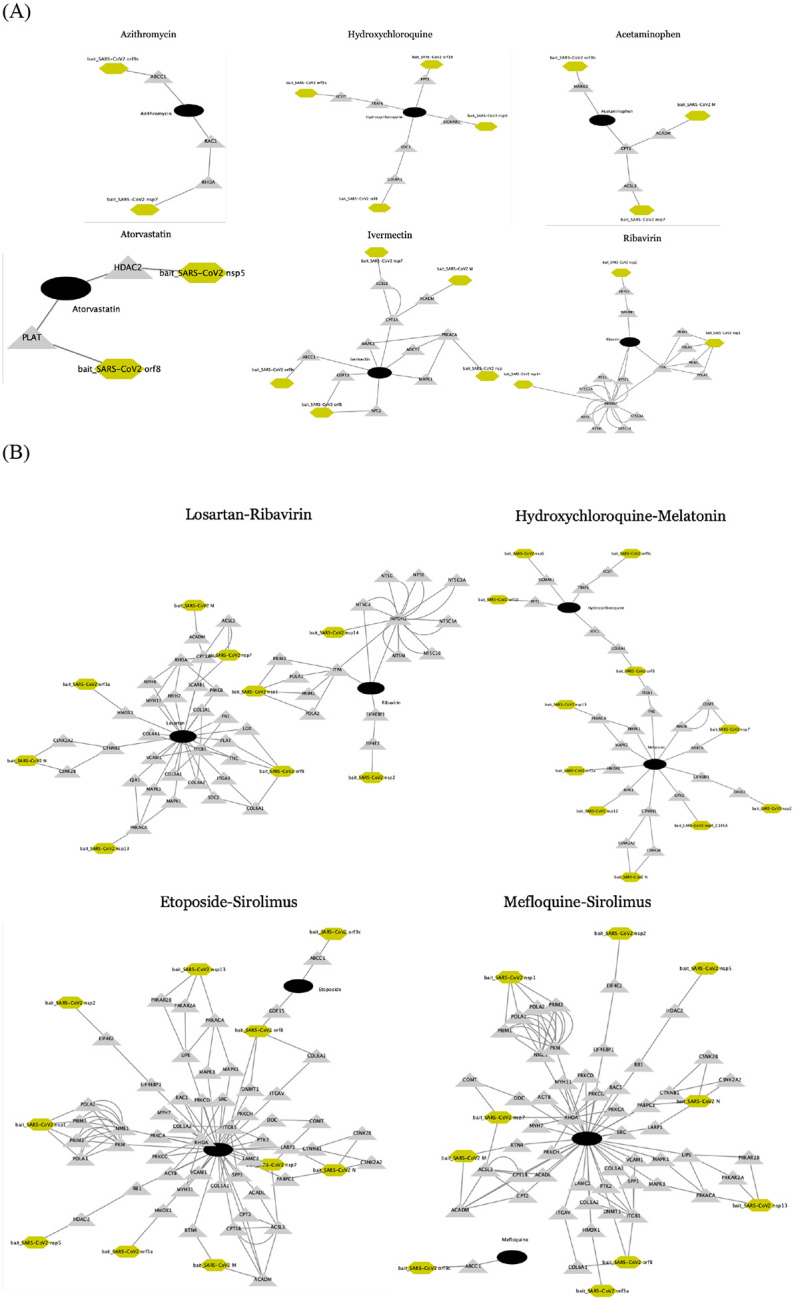
The interaction among virus baits, host preys, and drug targets. (a) single drugs (b) drug combinations. SARS-Cov-2 baits = green hexagons (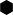). Genes = gray triangles (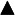) Drugs = black rounds (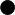). The potentially repurposable drugs directly and indirectly target the host gene, which has PPI with the virus baits.

Based on the extensive validation, we presented top repurposable drugs after filtering out and re-ordering the drug candidates according to the existence of validating evidence. We used a data programming technique to combine the multiple pieces of evidence (Note S3) (34). We highlighted the most promising drugs as follows (Fig. 3). Due to limited space, we presented the top 21 drugs in Table 4 and the remaining drugs are available in Table S3. The top 21 drugs include anti-infection, immunosuppressive or immunomodulatory, antiviral, anti-fever, antihypertensive, anti-cancer drugs, anticoagulant drugs which all have different possible functions in inhibiting SARS-CoV-2 proliferation or reducing symptoms. We highlight them below.

#### Antimicrobial Agents.

Azithromycin and Teicoplanin can inhibit 23 s ribosomes or RNA polymerase to stop the progress of infection. Some evidence supports Azithromycin regulating and/or decreasing the production of inflammatory mediators (IL-1β, IL-6, IL-8, IL-10, IL-12, and IFN-α), which might be effective to suppress viral entry (35). Azithromycin targets ABCC1 (an inflammatory modulator) that has direct PPI with SARS-CoV-2 bait orf9c (Fig. 3a). The data imply that Azithromycin can be related to viral gene replication. In the population-based EHR validation, Azithromycin had the highest treatment effect, and it is currently under testing in a clinical trial (36) to treat mild to moderate COVID-19 patients. Itraconazole can promote the production of IFN-1 that enhances viral-induced host responses (37).

#### Immunosuppressive drugs.

We identified immunosuppressive drugs such as Hydroxychloroquine, Chloroquine, and Sirolimus. Hydroxychloroquine or chloroquine are anti-parasite drugs but also have effects on toll-like receptors and ACE2 (38), where toll-like receptors are associated with the production of inflammatory mediators (IL-1, IL-6, TNF-α, IFN-α, and IFN-*β*) (39), and ACE2 is the entry receptor of SARS-CoV-2 (40). Hydroxychloroquine and chloroquine are rather controversial in terms of its effectiveness (41). . Hydroxychloroquine directly targets PPT1, SIGMAR1, TRAF6, and SDC1, and it indirectly targets ECSIT and COL6A1, which had PPIs with SARS-CoV-2 baits orf8, orf9c orf10, and nsp6 (Fig. 3a). Thus, hydroxychloroquine might interfere with the SARS-CoV-2 replication. Sirolimus also works on toll-like receptors (42).

#### Anti-fever drugs.

Aspirin inhibits COX1, COX2, and Acetaminophen inhibits COX3 (43). Acetaminophen directly targets ACADM, CPT2, and indirectly targets ACSL3, and MARK2 which finally have PPI with SARS-CoV-2 orf9b, M, and nsp7 (Fig. 3a). This means Acetaminophen may hinder the SARS-CoV-2 assembling and replication (44). Aspirin deactivates platelet function (45). A recent study reports that SARS-CoV-2 may over-activate platelets and thus reduce platelet production (46). Considering this evidence, Aspirin might be effective in COVID-19 patients by suppressing platelet function and inflammatory processes. Celecoxib is a COX2 selective inhibitor. According to a consensus docking result, Celecoxib inhibits SARS-CoV-2 main protease up to 37% (47). Celecoxib combined with Oseltamivir significantly reduces IL-6 and IL-10 and increases the survival rate of hospitalization (47).

#### Antiviral drugs.

We identified various antiviral drugs such as Remdesivir, Ribavirin, Lopinavir, and Tenofovir. Currently, Remdesivir has been proved to inhibit SARS-CoV-2 replication (48). In terms of PPI between the virus baits and host prey, Lopinavir targets HMOX1, which is a host prey that binds with SARS-CoV-2 orf3a (Fig. 3a). A recent study reports that Tenofovir may prevent SARS-CoV-2 replication (49). Ribavirin directly targets EIF4EBP1, IMPDH2, and TIPA, and it indirectly targets EIF4E2, POLA1, POLA2, PRM1, and PRM1, which have PPIs with SARS-CoV-2 baits nsp1, nsp2, and nsp14. This implies that Ribavirin may prevent SARS-CoV-2 replication.

#### Antihypertensive and Lipid-lowering drugs.

We identified Atorvastatin, Amlodipine, and Nifedipine. In addition to the original function for lowering cholesterol and triglyceride levels as an HMG-CoA reductase inhibitor, Atorvastatin can treat inflammation by lowering C-reactive protein (CRP) (50). Elevated CRP is highly associated with the aggravation of non-severe COVID-19 adult patients (51). Also, Atorvastatin targets PLAT, which is on the same regulatory pathway with HDAC2 (52), and HDAC2 is a host prey of the SARS-CoV-2 nsp5. The nsp5 can assist in releasing nsp4 and nsp16, which are involved in viral replication (27). Both Nifedipine and Amlodipine are calcium channel blockers. Nifedipine reduces the ACE2 expression (53) (54). In a retrospective study, Amlodipine prevents virus replication in COVID-19 (55).

#### Anti-cancer, Antipsychotic, and Hormone replacement drugs:

Isotretinoin, a Vitamin A derivative, binds to papain-like protease, an essential viral protein encoding by SARS-CoV-2 (56). Chlorpromazine, an antipsychotic drug shows an in-vitro efficacy in inhibiting viral entry of SARS-CoV-2 (56, 57). Progesterone decreases a severity of cytokine storms in COVID-19 patients (58).

### Drug combination search

As indicated by the complexity of the COVID-19 interaction network, using single drugs to treat the viral infection might result in short term effects. To improve treatment efficacy, we further predicted potential drug combinations from the top-ranking drugs with synergistic interactions without degradation in safety (71). Our working hypothesis was based on the Complementary Exposure pattern that “a drug combination is therapeutically synergistic if the targets of the individual drugs hit the disease module, but target a separate neighborhood” (72). We searched the drug combinations within the top 30 drugs. We highlight the potential drug combinations as below (Table 5, Fig. 3b).

#### Etoposide and Sirolimus.

Etoposide is an anti-cancer drug that targets DNA topoisomerase 2. Etoposide had been used to treat cytotoxic therapy for severe swine flu A/H1N1 (73). A recent report proposes that Etoposide can also suppress the inflammatory cytokines in COVID-19, by reducing activated cytotoxic T cells that further lead to eliminated activated macrophages (74). Sirolimus has been tested to be successful in treating MERS (75). There are some clinical trials to test the effectiveness of sirolimus in COVID-19 patients (NCT04341675). There is a clinical trial to test the effectiveness of combining Sirolimus, Celecoxib, and Etoposide on cancer (NCT02574728). Based on the virus bait-host prey interactome, this combination’ targets interact with ten virus baits (including orf9c, orf8, orf3a, nsp1, nsp2, nsp5) without overlapping targets. We can infer this combination can be related to virus assembly in mitochondria due to an association with nsp2 (27). The safety of this combination has been tested in treating Acute Myeloid Leukemia (76), but safety in COVID-19 still remains unknown.

#### Mefloquine and Sirolimus.

Mefloquine not only treats malaria but also has some effects on the immune system (77). The drug targets of Mefloquine and Sirolimus had similar baits-host prey interactome with Etoposide and Sirolimus.

#### Losartan and Ribavirin.

Losartan inhibits T-cell activation and also binds to ACE2 (78). Ribavirin has an antiviral and immunomodulatory function (79). From the bait-host gene PPI, this combination’s complementary drug targets had PPI with 9 virus baits including N, M, orf3a, orf8, nsp7, nsp1, nsp2, nsp13, and nsp14, which might affect the virus replication, assembling, and releasing (27).

#### Hydroxychloroquine and Melatonin.

Melatonin has been proposed as an adjuvant for COVID-19 treatment (80) because Melatonin can limit virus-related diseases with a high profile of safety. This might imply we can reduce the dosage of Hydroxychloroquine that decreases the risk of a long Q-T interval (38). This speculation needs further verification.

## DISCUSSION

The objective of this study is to prioritize repurposable drugs to treat COVID-19. We built the SARS-CoV-2 knowledge graph based on interactions from virus baits, host genes, pathways, drugs, and phenotypes. We then derived drug embedding using multi-relational graph neural representation and ranked drugs using the drug representation and the existence of clinical trial history. The drug ranking was validated from GSEA scores, in-vitro drug screening results, and COVID-19 hospitalized patients’ EHRs. As a result, our proposed pipeline prioritized Azithromycin, Atorvastatin, Acetaminophen, and Aspirin. Also, we identified drug combinations with complementary exposure patterns: Etoposide + Sirolimus, Mefloquine + Sirolimus, Losartan + Ribavirin, and Hydroxychloroquine + Melatonin.

Our contributions can be summarized as: i) integration of multiple and complementary perspectives from biological interactomes to genetic signatures, in-vitro efficacy, epidemiological effectiveness in EHRs, and clinical trial history; ii) methodological innovation to represent biological interaction using multi-relational graph neural networks and transfer learning; and iii) rigorously validated list of potentially repurposable drugs and their combination to treat COVID-19 that researchers can prioritize for further biological or clinical validation. Existing PPI-network-based studies (13, 27) use distance-based proximity scores and lack a deep understanding of overall topology in heterogeneous networks (i.e., multiple types of nodes). Our work utilized a deep graph neural network to overcome the barrier in representing extensive biological interactomes. A similar study that also uses knowledge graph representation for COVID-19 drug repurposing lacks extensive validation (15). Our work combined genetic, mechanistic, epidemiological validations to derive repurposable drugs that are not only statistically plausible but also biologically/clinically meaningful.

The main limitation of this study is the lack of statistical power in external validation. Although the wide spectrum of validation sources provided a complementary perspective, the statistical power of the accuracy was limited due to the small size of overlap between our initial drug set and the validation sources. Particularly in the population-based validation, 138 drugs overlapped between 3,635 initial drugs and 391drugs in EHRs. Only ten drugs (out of 138) were effective in the treatment effect analysis. Although our ranking model detected six drugs (out of ten) to be positive, the sample size is limited to obtain sufficient statistical power. As more data will be generated in the near future, we will further test and validate our approach and results.

We also observed conflicts across different validation sources. For example, Aspirin and Albuterol had positive treatment effects in EHRs validation, but there was no positive efficacy in all the four in-vitro experiments. Losartan was effective in GSEA but presents negative treatment effects in EHR validation. The reason for this discrepancy might be because each validation source captures different aspects of the drug’s function. The GSEA validation focused on inhibiting or activating the virus-associated host genes. The in-vitro efficacy focused on viral entry, replication, or cytopathic effect. The population-based EHRs validation focused on the drugs’ antiviral effect and also clinical symptom relief. For example, Acetaminophen, Azithromycin, and Albuterol are frequently given to hospitalized patients for fever, pneumonia, and shortness of breath, respectively. These drugs might not have a direct effect on the virus itself. Concordance in multiple validation sources may strengthen the confidence in the drug’s effectiveness. The drugs with conflicting validation results are still worth investigating.

We acknowledge limitations of noise or bias in the validation sources. The population-based validation was from observational and retrospective analyses of EHRs, which are inherently incomplete and erroneous compared to randomized experimental data. Our propensity score matching and weighting approach were designed to reduce bias and confounding effects, but unmeasured or hidden confounders may exist in the EHRs observational data. The other limitation is a discrepancy between gene sets from drug-induced gene expression and SARS-CoV-2-infected cell’s gene expression. cMAP provides the expression value for only 12,328 genes while the SARS-CoV-2-infected cell line (GSE153970) contains expression value for 17,899 genes. Consequently, the expression values for some genes in SARS-CoV-2 signature are missing, such as SARS-CoV2-gp10 and SARS-CoV2-gp01, which might cause bias. In spite of differences in cell line as well as missing expression value of some genes, the results still have some value as a reference for further investigation.

In conclusion, this study proposes an integrative drug repurposing pipeline for the rapid identification of drugs and their combination to treat COVID-19. Our pipelines were developed from extensive SARS-CoV-2 and drug interactions, deep graph neural representation, and ranking model, and validated from genetic profiles, in-vitro efficacy, and population-based EHRs. From a translational perspective, this pipeline can provide a general network pharmacology pipeline for various diseases, which can contribute to fast drug and drug combination repurposing (81).

## MATERIALS AND METHODS

### SARS-CoV-2 and human protein interactions

We collected the SARS-CoV-2 and host interaction data from a recent work that identifies 322 high confidence PPIs between SARS-CoV-2 and the human (27). This literature cloned 26 SARS-CoV-2 proteins in human cells and identified the human proteins that physically associated with the SARS-CoV-2 proteins. We used the SARS-CoV-2 and human protein interaction with MiST > 0.8. In total, the virus-host interaction network consisted of 27 virus baits and 332 SARS-CoV-2-associated prey proteins.

### Drug-target interactions

We collected drugs and targets from CTDbase’s COVID-19 curated list, which contains 5,065 potential targetable genes for COVID-19 with supporting biological mechanisms or therapeutic evidence. Potential compounds to SARS-CoV-2 were identified if the compounds target the SARS-CoV-2-associated genes. There were 3,635 compounds that target 4,427 genes. The size of the intersection between host genes interacting with baits and drug targets is 94.

### Biological pathways

We incorporated functional pathways related to SARS-CoV-2 infection and drugs of interest. We used the Kyoto Encyclopedia of Genes and Genomes (KEGG), Reactome (which were curated in CTDbase), and PharmGKB (24, 82). There were 1,763 unique genes and 13,423 pairs of genes that were associated with the pathways.

### Gene/drug-phenotype interactions

We used a curated set of phenotypes from CTDbase, which inferred the phenotypes via drug interaction and/or gene to gene ontology annotation. There were 1,285 phenotypes (i.e., biological process gene ontology) that were associated with 31 potential drugs and/or 18 SARS-CoV-2-associated genes.

### Embedding using graph neural network

We utilized deep graph neural embedding with multi-relational data (26). We used variational graph autoencoders with GraphSAGE message passing (20, 25). Due to uncertainty and incompleteness in our knowledge graph (i.e., COVID-19 is an emerging infectious disease and our knowledge on COVID-19 is developing), we chose to use variational autoencoders to account for the uncertainty. The graph autoencoder method is an unsupervised learning framework to encode the nodes into a latent vector (embedding) and reconstruct the given graph structure (i.e., graph adjacency matrix) with the encoded latent vector. The variational version of graph autoencoders is to learn the distribution of the graph to avoid overfitting during the reconstructing the graph adjacency matrix. In the message-passing step, each node (entity)’s embedding is iteratively updated by aggregating the neighbors embedding, in which the aggregation function is a mean of the neighbor’s features, concatenation with current embedding, and a single layer of a neural network on the concatenated one. We set different weight matrices for each of the five types of edges. Since our objective is to use the drug embedding to discover drugs that can functionally target SARS-CoV-2-associated host genes, the model was trained to reconstruct the missing interaction using the node embeddings as an unsupervised manner. We set the embedding size as 128 after several trials. We used PyTorch Geometric for implementation (83). The model structure was (1×400) → Graph convolution to (1×256) → RELU → Dropout → Concatenation of multiple edge types → Batch norm → Graph convolution to 1×128 (mean) and 1×128 (variance).

Our knowledge graph focused only on SARS-CoV-2-related baits, genes, drugs, and phenotypes. General biological interactions out of COVID-19 can benefit our learning process and enrich our embedding. To maximally utilize external databases out of COVID-19, we leveraged the Drug Repurposing Knowledge Graph (DRKG) (15), a large-scale comprehensive knowledge graph that represents the interaction between gene, drug, and related entities. We utilized the DRKG via transferring DRKG pre-trained node embedding (Note S1).

### Initial drug ranking

After we derived the drug embedding, we built a ranking model to select the most potent drugs. Drugs undergoing clinical trials were regarded as the first labels to identify drug candidates. The drugs under clinical trials were extracted from NIH ClinicalTrials.gov’s interventional trials (84). Ninety-nine trial drugs were matched to the CTDbase’s 3,635 drugs. We designed a simple neural-network-based ranking model with Bayesian pairwise ranking loss (29, 85). The architecture was two fully connected layers (with the size of 128→128→1) with residual connection, nonlinear activation (ReLU), dropout, batch norm in the middle, and the optimization loss (Bayesian pairwise ranking loss). Baseline ranking models to compare were logistic regression, support vector machine, XGBoost, and Random forest.

We measured the accuracy of the drug ranking model using the area under the receiver operating curve (AUROC) and area under the precision-recall curve (AUPRC) with 50% training and 50% test cross-validation. We purposely set the portion of the training set lower because the clinical trials are not our sole “gold standard” to prioritize drugs. Note that the unsupervised knowledge graph embedding and the supervised drug ranking were independent. We tried to avoid using the supervised label (clinical trials drugs) in the knowledge graph embedding because the drugs being considered in clinical trials do not guarantee the efficacy of the drugs.

### Genetic validation using gene set enrichment analysis

For the genetic validation, we evaluated the drugs by calculating GSEA scores between gene expression profiles of SARS-CoV-2-infected host cells and the gene signature of the drugs. The SARS-CoV-2 genetic profile was three samples from SARS-CoV-2 infected primary human airway epithelial cell lines and three mock-infected (PBS) cell lines (GSE153970). *Deseq2* was used to detect the differentially expressed genes (DEGs) by adjusted *p*-value less than 0.01 (86). The up-regulated and down-regulated genes from DEGs were considered as an up-regulated SARS-CoV-2 signature and down-regulated SARS-CoV-2 signature. The drug’s genetic profiles were obtained from the drug-induced gene expression in cMAP (GSE92742 and GSE70138) (45). The whole drugs’ gene probe set was ordered from the highest up-regulated genes to the lowest down-regulated genes.

The enrichment score (ES) was calculated to reflect the correlation between the SARS-CoV-2 signature and the drug’s gene expression (43) by connectivity map algorithms (32). The hypothesis was that if the drug’s gene expression is opposite with the disease up-regulated or down-regulated signature, the drug tends to treat disease (87). ES is calculated as follows (33):

$$ES=(ES_{up}-ES_{down})∕2\;if\;sgn(ES_{up})\neq sgn(ES_{down});\;else\;0.$$

*ES_up_* is the enrichment score for SARS-CoV-2 up-regulated signature; *ES_down_* is the enrichment score for SARS-CoV-2 down-regulated signature. If *ES_up_*and *ES_down_* have the same algebraic sign, the value of final ES is set to 0. Otherwise, ES is the difference between *ES_up_*and*ES_down_*. *ES_down_*and *ES_down_* was calculated based on the weighted Kolmogorov-Smirnov enrichment statistic (ES) (30). In order to obtain *p*-value, permutation tests were done by randomly generating the same number of genes as upregulated gene set and downregulated gene set separately and thus we can get the null distribution of random ES. We identified a significant genetic association between the drug and the disease by setting a threshold as ES < 0 and *p*-value <0.05, which means a drug has opposite effects for both up-regulated SARS-CoV-2 (*ES_up_* < 0) and down-regulated SARS-CoV-2 set (*ES_down_* > 0).

### Retrospective in vitro drug screening validation

We validated the highly ranked candidate drugs by retrospectively comparing them with efficacious drugs in multiple in vitro drug screening studies. We utilized 4 drug screening studies targeting viral entry (ACE2 enzymatic activity, Spike-ACE2 protein-protein interaction) and viral replication/infection (cytopathic effect), which are obtained from NCATS OpenData COVID-19 Portal and Riva et al. study (5, 6). The two viral entry assay studies screened 2,678 compounds in the NCATS Pharmaceutical Collection and 739 compounds in the NCATS Anti-infectives Collection (88). In the viral entry assay, a drug was regarded as efficacious if efficacy value was larger than 10 and 0 for ACE2 enzymatic activity and Spike-ACE2 interaction, respectively (the efficacy value was defined as an % inhibition at infinite concentration subtracted by % inhibition at zero concentration by curve fitting). The two cytopathic effects studies use either the NCATS collections or the ReFRAME drug library on the same Vero E6 cell (89). In the NCATS cytopathic effect study, a drug was regarded as efficacious if the efficacy value was larger than 10. In the ReFRAME study, a drug was regarded as efficacious if the drug inhibited infection by 40% or more (5).

### Population-based validation

We also conducted the population-level counterfactual analysis for candidate drugs on treating COVID-19 using EHRs (non-experimental data, as opposed to randomized clinical trials). The key is to reduce bias or confounders in EHRs to control the difference of confounding variables between those who received and did not receive treatment. We calculated the average treatment effect on the treated (ATT) using propensity score matching and weighting (Note S2).

In this study, we used EHRs with 140,016 positive COVID-19 patients. There were a total of 34,043 hospitalized COVID-19 patients, we selected 3,200 deceased patients during the hospitalization and 15,078 recovered patients with medication history and length of stay > 2 days. From the selected hospitalized patients, we built a cohort with 2,827 cases (deceased) and 2,774 controls (recovered) that follow similar distributions in terms of demographics (race, ethnicity, sex, age) and admission severity (body temperature and SPO_2_) using PSM. After we derived the matched cohort, there were a total of 391 medications that were administered in at least 35 patients. We calculated the treatment effect of the 391 medications using the average treatment effect among treated or ATT. For the inverse propensity score weighting, we considered demographics (age, gender, race), admission conditions (body temperature, SPO_2_), comorbidities (cancer, chronic kidney disease, obesity, a serious heart condition, solid organ transplant, COPD, type II diabetes, and sickle cell disease), and drug history before the treatment of interest. We assumed a drug is effective if ATT>0 and the *p*-value is <0.05. A full list of the drug's ATT coefficient is in Table S2.

### Drug combination search

We identified efficacious drug combinations from top-ranked drugs. Our approach is to leverage drug targets and COVID-19 associated host genes. Our hypothesis was that “a drug combination is therapeutically effective only if the targets of the drugs both hit the disease module, but they target a separate neighborhood (Complementary Exposure pattern)” (72). We identified the COVID-19 modules from human protein interactomes that are physically associated with SARS-CoV-2 baits (27). The drug’s targets were identified from CTDbase’s COVID-19 curated list (90). We counted the number of genes in the COVID-19 module that a drug combination hits, where the drug combination’s targets are disjoint.

## Supplementary Material

Supplement Supplementary Table S1. Link prediction accuracy in the SARS-CoV-2 knowledge graph.
Supplementary Table S2. ATT score of 138 drugs that were in EHRs and initial 3,635 drugs.
Supplementary Table S3. Full list of repurposable drugs
Supplementary Table S4. Full list of drug combinations from the top drugs.

Supplement Supplementary Figure S1. The SARS-CoV-2 Knowledge Graph
Supplementary Figure S2. Interactive t-sne plot
Supplementary Figure S3. External validation (a) Accuracy was measured in the intersection of candidate drugs and external validation sources. (b) Cohort selection and propensity score matching in EHRs.

Supplement Supplementary Note S1 Transfer learning
Supplementary Note S2 Calculating the treatment effect
Supplementary Note S3 Re-ordering the validated drugs
Supplementary Note S4 Genetic validation using gene set enrichment analysis

Supplement

## Figures and Tables

**Table 1.** Accuracy of predicting drugs under COVID-19 clinical trials. The predictors were the drug embedding and labels that were whether a drug is under clinical trials. Logistic Regression, Support Vector Machines, XGBoost, and Random Forest were off-the-shelf models. The neural network is a customized model (Methods). AUROC=area under the receiver operating curve. AUPRC=area under the precision-recall curve.

Embedding Methods	Accuracy	Ranking models				
		Logistic Regression	Support Vector Machines	XGBoost	Random Forest	Neural network ranking
SARS-CoV-2 knowledge graph embedding	AUROC	0.6800	0.6915	0.7019	0.6161	0.7628
	AUPRC	0.0604	0.1149	0.0836	0.0940	0.1272
General biomedical knowledge graph embedding from DRKG (15)	AUROC	0.7855	0.8332	0.8500	0.7372	0.8512
	AUPRC	0.1183	0.1848	0.1439	0.0790	0.1624
**SARS-CoV-2 knowledge graph embedding + general embedding (proposed)**	AUROC	0.8973	0.7697	0.8934	0.7814	0.8992
	AUPRC	0.1965	0.1629	0.1701	0.0916	0.2503

**Table 2.** External validation of the candidate drugs using in vitro drug screening results and EHRs. N/A=not available. False-negative or true-negative values could not be obtained because the cytopathic effect (ReFRAME) study only reports positive drugs (5). Caution is needed in interpreting the accuracy because the number of overlapping drugs is limited in some studies and, thus, the statistical power is limited.

Validation type	Source	# overlap drugs	# true positives (TP)	# false positives (FP)	# false negatives (FN)	# true negatives (TN)	Recall TP/(TP +FP)	Precision TP/(TP+F N)
Gene profiles	GSEA scores	580	55	128	128	269	0.3006	0.3006
In-vitro drug screening results	ACE2 enzymatic activity (6)	497	25	69	120	283	0.2660	0.1724
	Spike-ACE2 protein-protein interaction (6)	497	6	22	139	330	0.2143	0.0414
	Cytopathic effect (NCATS) (6)	497	26	33	119	319	0.4407	0.1793
	Cytopathic effect (ReFRAME) (5)	13	5	8	N/A	N/A	0.3846	N/A
Population based	EHRs	138	6	4	52	76	0.6	0.1035

**Table 3.** COVID-19 hospitalized patient’s demographics and comorbidities before and after PSM.

	Before matching		After matching	
	Recovered	Deceased	Recovered	Deceased
Number of patients	15,078	3,200	2,774	2,827
Age				
Mean	60.10	73.78	73.64	73.24
Standard deviation	17.63	12.81	12.95	12.86
Sex				
Male	7,765	1,887	1,601	1,630
Female	7,309	1,313	1,172	1,197
Race				
Caucasians	7336	2031	1728	1790
African Americans	4052	544	511	490
Asian Americans	470	113	97	102
Others	3,220	512	438	445
Admission conditions				
Temperature	36.93	37.16	37.07	37.00
SPO_2_	94.21	91.39	92.32	92.56

**Table 4.** Top 22 promising drugs with supporting evidence and literature. + : positive evidence, −: negative evidence, NA: not investigated. Positive in-vitro efficacy if there is at least one positive efficacy in the four different in-vitro experiments. Full list in Table S3.

Drug name	Treated for	Targets	GSEA score	In-vitro efficacy	EHRs	Clinica l trials	Supporting literature
Azithromycin	Anti-infection	23 s ribosome of bacteria	+	+	+	+	(35)
Hydroxychloroquine	Immunosuppressive drug, Anti-parasite	TLR-7, TLR-9, ACE2	NA	+	+	+	(59)
Atorvastatin	Lipid-lowering	HMG-CoA Inhibitor	+	NA	+	+	(60)
Acetaminophen	Pain, fever	PGE-3, COX-1, COX-2	NA	+	+	+	NA
Aspirin	Pain, fever	COX-1, COX-2	−	−	+	+	NA
Albuterol	Anti-asthma	beta-2-agonist	NA	−	+	−	NA
Melatonin	Sleep awake cycle	Melatonin receptor	+	+	−	+	(61)
Sirolimus	Immunomodulatory	mTOR	+	−	NA	+	(62)
Nifedipine	Anti-hypertension	Calcium channel	+	+	−	+	(63)
Ribavirin	Anti-HCV	IMP-synthesis	NA	+	NA	+	(64)
Chloroquine	Immunosuppressive drug, Anti-parasite	TNF, TLR-9, ACE2	NA	+	NA	+	(38, 59)
Lopinavir	Anti-HIV	HIV-protease	NA	+	NA	+	(63)
Teicoplanin	Anti-infection	peptidoglycan	NA	+	NA	+	(65)
Remdesivir	Ebola, COVID-19	RNA polymerase	NA	+	−	+	(66)
Ivermectin	Anti-parasite	Glycine receptor subunit alpha-3	NA	+	NA	+	(67)
Amlodipine	Anti-hypertension	Calcium channel	+	+	−	+	(47, 61)
Celecoxib	Anti-inflammatory	CoX2	+	+	NA	+	(47)
Isotretinoin	Anti-cancer	Vitamin A derivative	+	+	NA	+	(68)
Chlorpromazine	Antipsychotic	D1/D2 receptor	+	+	NA	+	(69)
Itraconazole	Anti-fungus	Lanosterol 14-alpha demethylase	+	+	NA	+	(37)
Progesterone	Hormone replacement	Progesterone receptor	+	+	NA	+	(58)
Tenofovir	Anti-HIV	Reverse transcriptase	+	NA	NA	+	(70)

**Table 5.** Drug combinations that satisfy the complementary exposure pattern from the top 30 drugs (72). COVID-19 genes were defined as the host genes that have PPIs with SARS-CoV-2 baits. The full list in Table S4.

Drug A	Drug B	# COVID-19 genes that Drug A hits	# COVID-19 genes that Drug B hits	# COVID-19 genes that either Drug A or B hit
Etoposide	Sirolimus	2	22	24
Mefloquine	Sirolimus	1	22	23
Losartan	Ribavirin	12	6	18
Hydroxychloroquine	Melatonin	4	10	14
Etoposide	Losartan	2	12	14
Acetaminophen	Chloroquine	3	11	14
Losartan	Mefloquine	12	1	13
Chloroquine	Lopinavir	11	2	13
Chloroquine	Atorvastatin	11	2	13
Acetaminophen	Melatonin	3	10	13
