# Supplementary material for: Drug Repurposing for COVID-19 using Graph Neural Network with Genetic, Mechanistic, and Epidemiological Validation
Source: Res Sq. 2020 Dec 11:rs.3.rs-114758. Preprint. [Version 1] doi: 10.21203/rs.3.rs-114758/v1 (PMC7743080; doi:10.21203/rs.3.rs-114758/v1)
Supplement: Supplement — ary Table S1. Link prediction accuracy in the SARS-CoV-2 knowledge graph. Supplementary Table S2. ATT score of 138 drugs that were in EHRs and initial 3,635 drugs. Supplementary Table S3. Full list of repurposable drugs Supplementary Table S4. Full list of drug combinations from the top drugs. [file 15a118317ad785e0ef173cac.pdf]

Supplementary Table S1. Link prediction accuracy in the SARS-CoV-2 knowledge graph.

	AUROC	AUPRC
SARS-CoV-2 knowledge graph embedding	0.8132	0.8536
General embedding (*17*)	0.5695	0.6431
SARS-CoV-2 knowledge graph embedding + general embedding (proposed)	0.8121	0.8524

Supplementary Table S2. ATT score of 138 drugs that were in EHRs and initial 3,635 drugs.

Drug Name	Propensity score weighting		
	pvalue (ATT)	t-stats (ATT)	treatment coefficient (ATT)
ACETAMINOPHEN	1.52E-24	10.2738974	0.25207263
CEFTRIAXONE	2.28E-38	13.0506026	0.21025986
AZITHROMYCIN	1.47E-38	13.0848348	0.18160471
HEPARIN	0.49937998	0.67550956	0.02587599
ATORVASTATIN	0.00650385	2.72225646	0.17
ALBUTEROL	2.94E-08	5.55278765	0.14218626
ASPIRIN	0.02040798	2.3194218	0.13577879
LISINOPRIL	0.01856643	2.35481156	0.08691948
METOPROLOL	0.33616909	-0.9618448	-0.0304113
HYDROXYCHLOROQUINE	0.00145871	3.18435704	0.07652113
GLUCAGON	9.86E-06	4.42425963	0.07651207
METHYLPREDNISOLONE	0.32935996	-0.9754889	-0.0576644
AMLODIPINE	0.08001674	1.75090671	0.13155142
NALOXONE	0.02934147	2.17942738	0.07071353
MELATONIN	0.33434018	0.96549192	0.03443055
VANCOMYCIN	0.00716502	-2.6900641	-0.0792951
FUROSEMIDE	0.01608931	-2.4076136	-0.0872555
CLOPIDOGREL	0.01611531	-2.4070233	-0.1028589
ASCORBIC ACID	0.48643081	-0.696043	-0.0322171
DOXYCYCLINE	0.81055864	0.23971674	0.00682517
LIDOCAINE	0.55576054	-0.5891856	-0.019582
HYDRALAZINE	0.88880536	-0.1398224	-0.0070413
FAMOTIDINE	0.00734453	-2.6817855	-0.1079076
LIDOCAINE	0.07692957	-1.7691123	-0.0624069
AMIODARONE	0.02153417	-2.2991305	-0.1086655
METOPROLOL	0.17138449	1.36794405	0.04936244
CHOLECALCIFEROL	0.10593916	-1.616979	-0.0543447
IOHEXOL	0.72538086	-0.3512942	-0.014653
PREDNISONE	0.48818378	0.69324625	0.07798455
CEFEPIME	0.00568527	-2.7664702	-0.1139592
DEXAMETHASONE	0.0338526	-2.1223445	-0.1244167
ATROPINE	0.02212069	-2.2889265	-0.1303156
DIPHENHYDRAMINE	0.64771518	-0.4569633	-0.0291316
NITROGLYCERIN	0.04374195	-2.0170115	-0.1419787
FINASTERIDE	0.01168076	-2.5224736	-0.144157
LABETALOL	0.86450268	-0.1706531	-0.0113445

LOSARTAN	0.81224008	0.23754839	0.01065086
HYDROCORTISONE	0.00010421	-3.883361	-0.1530126
CARVEDILOL	0.24598449	-1.1602795	-0.0551179
HYDROCHLOROTHIAZIDE	0.03735116	-2.0824009	-0.161645
OLANZAPINE	0.10617452	-1.6158896	-0.092137
METOCLOPRAMIDE	0.85496239	-0.182799	-0.0132935
LACTULOSE	0.36285386	-0.9100148	-0.0587638
DEXMEDETOMIDINE	7.05E-05	-3.9776907	-0.1694607
IOPAMIDOL	0.01013427	-2.572088	-0.173839
SERTRALINE	0.00581868	-2.7588881	-0.1776973
HALOPERIDOL	3.75E-07	-5.0871542	-0.1851163
PRAVASTATIN	0.03642423	-2.0926622	-0.1874756
FOLIC ACID	0.00038414	-3.5529034	-0.1900155
IBUPROFEN	0.00813824	-2.6472261	-0.1995362
LIDOCAINE	0.5169987	0.64801983	0.06157075
KETAMINE	0.0086921	-2.6248566	-0.2015791
TRAMADOL	0.63851705	0.46979882	0.02232213
METRONIDAZOLE	0.283333	-1.0729651	-0.0623913
LOPERAMIDE	0.75375249	-0.3137107	-0.0302894
SIMVASTATIN	0.3622305	-0.9111976	-0.0554117
MONTELUKAST	0.01230472	-2.5040952	-0.2107523
DONEPEZIL	0.00700435	-2.6976335	-0.212248
PHENYLEPHRINE	2.15E-08	-5.6079682	-0.2196067
FENTANYL	0.00893363	-2.6154986	-0.2201461
NYSTATIN	0.32781182	-0.9786164	-0.0510907
ALPRAZOLAM	0.00041075	-3.5352067	-0.2220222
WARFARIN	0.41192227	-0.8205769	-0.0467396
DIGOXIN	0.07103709	-1.8055836	-0.126615
REMDESIVIR	0.15411194	-1.4253495	-0.138926
DOBUTAMINE	0.01213208	-2.5090958	-0.2344604
BENZOCAINE	0.55839402	-0.5852637	-0.0653141
CETIRIZINE	0.04359487	-2.0184225	-0.2386508
ACETYLCYSTEINE	0.43246961	-0.7850293	-0.0519943
EPINEPHRINE	0.00759383	-2.670584	-0.240865
FENTANYL	8.13E-13	-7.1756851	-0.2492124
ACETAZOLAMIDE	1.86E-06	-4.7733488	-0.2617033
OMEPRAZOLE	0.07503783	-1.7805645	-0.1258121
BUDESONIDE	0.00197658	-3.0951894	-0.2667685
CLONAZEPAM	0.73848942	0.33387099	0.03418501
ARIPIPRAZOLE	0.03403606	-2.1201643	-0.2671175

DIAZEPAM	0.46286609	-0.7341861	-0.0588237
CLONIDINE	0.3939126	0.85260908	0.07337225
ADENOSINE	0.00628353	-2.7336401	-0.2767434
MORPHINE	3.17E-16	-8.1912788	-0.2768542
RIVAROXABAN	0.00296652	-2.9724904	-0.2769312
HEPARIN	0.54654473	-0.6029825	-0.0927038
LEVOFLOXACIN	1.11E-05	-4.3983949	-0.2910326
PROPOFOL	2.91E-16	-8.2020135	-0.2935477
NOREPINEPHRINE	2.89E-06	-4.6832344	-0.3247254
NIFEDIPINE	0.83811023	0.20432082	0.04712296
DEXTROMETHORPHAN	0.98456441	-0.0193477	-0.003308
METFORMIN	0.17324854	1.36201312	0.12826838
TORSEMIDE	0.21301729	-1.2454533	-0.1046479
SCOPOLAMINE	2.15E-06	-4.7434449	-0.3333922
ATENOLOL	0.00113185	-3.2572062	-0.3503093
MORPHINE	0.03087905	-2.1591735	-0.3537295
VANCOMYCIN	0.00964435	-2.5892168	-0.3567775
MEMANTINE	0.00121615	-3.236721	-0.3594972
FENOFIBRATE	0.04108996	-2.0430929	-0.369644
NICARDIPINE	0.52863581	-0.6301291	-0.0696733
ROPINIROLE	0.02902722	-2.1836789	-0.4452383
SUCRALFATE	0.64712573	-0.4577835	-0.075371
SPIRONOLACTONE	2.00E-05	-4.2682286	-0.4612046
HYDROXYCHLOROQUINE	0.00945421	-2.5960739	-0.4639617
GLUCAGON	0.01868063	-2.3525295	-0.4807472
HYDROCORTISONE	0.84177018	0.19963899	0.02369427
PROMETHAZINE	0.00011186	-3.8660631	-0.5801215
NICOTINE	0.09910498	-1.6494822	-0.2238539
FLUOXETINE	1.06E-06	-4.8853352	-0.6123011
VALSARTAN	0.15097006	-1.4363099	-0.1778135
HYDROXYZINE	0.63969501	-0.4681507	-0.090603
CIPROFLOXACIN	0.85665827	0.18063799	0.04088519
EZETIMIBE	7.27E-05	-3.9701899	-0.6325472
ARGATROBAN	0.32316911	-0.9880536	-0.1438822
BACLOFEN	3.15E-05	-4.166052	-0.6328379
CEFTRIAXONE	0.00040868	-3.5365442	-0.6675039
CITRIC ACID	0.21153297	-1.2495041	-0.1447582
VANCOMYCIN	2.04E-08	-5.6169419	-0.6738155
NITROGLYCERIN	0.70381577	-0.3801941	-0.0926956
PREGABALIN	2.75E-05	-4.196547	-0.6965285

METHYLPREDNISOLONE	0.94484106	-0.0691897	-0.0266676
OSELTAMIVIR	3.40E-05	-4.148281	-0.976161
DEXAMETHASONE	0.57765648	-0.5568437	-0.1242039
TACROLIMUS	2.29E-05	-4.2377794	-1.0332676
CALCITRIOL	9.04E-07	-4.9170129	-1.0514741
BARIUM	0.22893864	1.20322888	0.1784027
DOPAMINE	0.40608679	-0.8308624	-0.0840319
PHENOBARBITAL	2.85E-12	-7.0007173	-1.0735688
CLONIDINE	0.88588016	-0.1435257	-0.0265769
COLCHICINE	0.68956156	0.39947082	0.08690109
LANSOPRAZOLE	0.27627731	-1.0888264	-0.1837033
WARFARIN	0.4582038	-0.7418592	-0.2969037
PAROXETINE	0.39760705	-0.8459675	-0.1215711
PROPRANOLOL	0.06323556	-1.8579008	-0.3826055
VALPROIC ACID	0.83680638	-0.2059898	-0.0392783
HEPARIN	9.87E-08	-5.336122	-1.1812906
PHENOL	0.32108257	-0.9923236	-0.2197819
VERAPAMIL	0.98816939	0.01482867	0.00583047
AMITRIPTYLINE	0.94666174	0.06690246	0.02770827
FENTANYL	0.00028005	-3.6353914	-1.3469621
ALBUTEROL	0.00015378	-3.7874736	-1.6366453
TRIAMCINOLONE ACETONIDE	0.86991748	-0.1637708	-0.0429702

Supplementary Table S3. Full list of repurposable drugs

Drug	Initial rank	Initial rank within top 30	GSEA (1 if ES<0, p-value< 0.05)	ACE2 enzymatic activity	Spike-ACE2 protein-protein interaction (AlphaLISA)	SARS-CoV-2 cytopathic effect (NCATS)	SARS-CoV-2 cytopathic effect (ReFRAME)	Positive efficacy in any in-vitro experiments	EHR	Under_trials	Prob
Azithromycin	20	1	1	-1	-1	1	0	1	1	1	0.9998
Hydroxychloroquine	23	1	0	0	0	0	0	0	1	1	0.9988
Atorvastatin	44	0	1	0	0	0	0	0	1	1	0.9979
Acetaminophen	75	0	0	1	-1	-1	0	1	1	1	0.9978
Aspirin	189	0	-1	-1	-1	-1	0	0	1	1	0.9899
Albuterol	270	0	0	0	0	0	0	0	1	0	0.9802
Melatonin	13	1	1	-1	-1	-1	0	0	-1	1	0.9786
Sirolimus	21	1	1	-1	-1	-1	0	0	0	1	0.9786
Nifedipine	9	1	1	-1	-1	-1	0	0	-1	1	0.9786
Ribavirin	22	1	0	-1	1	-1	0	1	0	1	0.9777
Chloroquine	15	1	0	-1	-1	1	1	1	0	1	0.9777
Lopinavir	14	1	0	-1	-1	1	0	1	0	1	0.9777
Teicoplanin	0	1	0	1	-1	1	0	1	0	1	0.9777
Remdesivir	7	1	0	-1	-1	1	1	1	-1	1	0.9777
Ivermectin	11	1	0	-1	-1	1	0	1	0	1	0.9777
Amlodipine	248	0	1	-1	-1	1	0	1	-1	1	0.9624
Celecoxib	55	0	1	1	-1	1	0	1	0	1	0.9624
Isotretinoin	60	0	1	1	-1	-1	0	1	0	1	0.9624
Chlorpromazine	182	0	1	-1	-1	1	0	1	0	1	0.9624
Itraconazole	34	0	1	-1	-1	1	0	1	0	1	0.9624
Progesterone	32	0	1	-1	-1	1	0	1	0	1	0.9624
Tenofovir	12	1	0	-1	-1	-1	0	0	0	1	0.9416
Mefloquine	1	1	0	0	0	0	0	0	0	1	0.9416
Ritonavir	4	1	0	-1	-1	-1	0	0	0	1	0.9416
Heparin	30	1	0	0	0	0	0	0	-1	1	0.9416
Cyclosporine	29	1	0	0	0	0	0	0	0	1	0.9416
Etoposide	25	1	0	-1	-1	-1	0	0	0	1	0.9416
Losartan	19	1	0	-1	-1	-1	0	0	-1	1	0.9416

Toremifene	2	1	1	0	0	0	0	0	0	0	0.9324
Cepharanthine	3	1	0	-1	-1	1	1	1	0	0	0.9298
Arbidol	5	1	0	-1	-1	1	0	1	0	0	0.9298
Valsartan	43	0	1	-1	-1	-1	0	0	-1	1	0.9038
Yohimbine	100	0	1	-1	-1	-1	0	0	0	1	0.9038
Clopidogrel	246	0	1	0	0	0	0	0	-1	1	0.9038
Naltrexone	136	0	1	0	0	0	0	0	0	1	0.9038
Amoxicillin	38	0	1	0	0	0	0	0	0	1	0.9038
Simvastatin	95	0	1	-1	-1	-1	0	0	-1	1	0.9038
Leflunomide	156	0	1	-1	-1	-1	0	0	0	1	0.9038
Thalidomide	175	0	1	-1	-1	-1	0	0	0	1	0.9038
Fluoxetine	103	0	1	0	0	0	0	0	-1	1	0.9038
Dexamethasone	80	0	1	-1	-1	-1	0	0	-1	1	0.9038
Lenalidomide	267	0	1	-1	-1	-1	0	0	0	1	0.9038
Eicosapentaenoic Acid	125	0	0	1	-1	-1	0	1	0	1	0.9003
Melphalan	127	0	0	1	-1	-1	0	1	0	1	0.9003
Famotidine	173	0	0	1	-1	-1	0	1	-1	1	0.9003
Omeprazole	91	0	0	1	-1	-1	0	1	-1	1	0.9003
Trimethoprim	112	0	0	1	-1	-1	0	1	0	1	0.9003
Dexmedetomidine	285	0	0	1	-1	-1	0	1	-1	1	0.9003
Clozapine	62	0	1	1	-1	1	0	1	0	0	0.8853
Tamibarotene	114	0	1	-1	-1	-1	1	1	0	0	0.8853
Tamoxifen	81	0	1	-1	-1	1	0	1	0	0	0.8853
Clonidine	275	0	1	1	-1	-1	0	1	-1	0	0.8853
Troglitazone	83	0	1	1	-1	1	0	1	0	0	0.8853
Ranitidine	162	0	1	1	-1	-1	0	1	0	0	0.8853
Fenofibrate	281	0	1	1	-1	-1	0	1	-1	0	0.8853
Tretinoin	56	0	1	0	0	0	1	1	0	0	0.8853
Amphetamine	28	1	0	0	0	0	0	0	0	0	0.8295
Gentamicins	27	1	0	0	0	0	0	0	0	0	0.8295
Doxorubicin	26	1	0	-1	-1	-1	0	0	0	0	0.8295
Valproic Acid	24	1	0	0	0	0	0	0	-1	0	0.8295
Betulinic Acid	18	1	0	-1	-1	-1	0	0	0	0	0.8295

Dactinomycin	17	1	0	0	0	0	0	0	0	0	0.8295
Antiviral Agents	16	1	0	0	0	0	0	0	0	0	0.8295
Emodin	10	1	0	0	0	0	0	0	0	0	0.8295
Immucillin A	8	1	0	0	0	0	0	0	0	0	0.8295
(2-Tert-Butoxy-1-(2-Cyclohexyl-1-(1-Formyl-2-(2-Oxopyrrolidin-3-Yl)Ethylcarbamoyl)Ethylcarbamoyl)Propyl)Carbamic Acid Benzyl Ester	6	1	0	0	0	0	0	0	0	0	0.8295
Tacrolimus	36	0	0	0	0	0	0	0	-1	1	0.7683
Quercetin	92	0	0	-1	-1	-1	0	0	0	1	0.7683
Hydrocortisone	98	0	0	-1	-1	-1	0	0	-1	1	0.7683
Methotrexate	84	0	0	-1	-1	-1	0	0	0	1	0.7683
Naproxen	120	0	0	0	0	0	0	0	0	1	0.7683
Folic Acid	82	0	0	-1	-1	-1	0	0	-1	1	0.7683
Nitric Oxide	115	0	0	0	0	0	0	0	0	1	0.7683
Candesartan	48	0	0	-1	-1	-1	0	0	0	1	0.7683
Iloprost	274	0	0	-1	-1	-1	0	0	0	1	0.7683
Spironolactone	53	0	0	-1	-1	-1	0	0	-1	1	0.7683
Doxycycline	222	0	0	-1	-1	-1	0	0	-1	1	0.7683
Isoflurane	219	0	0	-1	-1	-1	0	0	0	1	0.7683
Oxygen	45	0	0	0	0	0	0	0	0	1	0.7683
Vitamin A	264	0	0	0	0	0	0	0	0	1	0.7683
Metformin	263	0	0	-1	-1	-1	0	0	-1	1	0.7683
Bicalutamide	201	0	0	-1	-1	-1	0	0	0	1	0.7683
Oseltamivir	203	0	0	0	0	0	0	0	-1	1	0.7683
Prednisolone	210	0	0	-1	-1	-1	0	0	0	1	0.7683

Estradiol	57	0	0	-1	-1	-1	0	0	0	1	0.7683
Propofol	214	0	0	-1	-1	-1	0	0	-1	1	0.7683
Amiodarone	218	0	0	0	0	0	0	0	-1	1	0.7683
Ibuprofen	191	0	0	0	0	0	0	0	-1	1	0.7683
Alitretinoin	130	0	1	0	0	0	0	0	0	0	0.7392
Temozolomide	240	0	1	-1	-1	-1	0	0	0	0	0.7392
Bosentan	277	0	1	-1	-1	-1	0	0	0	0	0.7392
Bezafibrate	245	0	1	-1	-1	-1	0	0	0	0	0.7392
Riluzole	236	0	1	-1	-1	-1	0	0	0	0	0.7392
Sumatriptan	110	0	1	-1	-1	-1	0	0	0	0	0.7392
Levofloxacin	283	0	1	-1	-1	-1	0	0	-1	0	0.7392
Naringenin	284	0	1	0	0	0	0	0	0	0	0.7392
Propranolol	137	0	1	0	0	0	0	0	-1	0	0.7392
Triprolidine	149	0	1	-1	-1	-1	0	0	0	0	0.7392
Gemfibrozil	225	0	1	-1	-1	-1	0	0	0	0	0.7392
Theophylline	154	0	1	0	0	0	0	0	0	0	0.7392
Montelukast	170	0	1	0	0	0	0	0	-1	0	0.7392
Propylthiouracil	172	0	1	-1	-1	-1	0	0	0	0	0.7392
4-Methyl-N1-(3-Phenylpropyl)Benzene-1,2-Diamine	205	0	1	0	0	0	0	0	0	0	0.7392
Mercaptopurine	194	0	1	-1	-1	-1	0	0	0	0	0.7392
Glyburide	299	0	1	0	0	0	0	0	0	0	0.7392
Carbamazepine	297	0	1	-1	-1	-1	0	0	0	0	0.7392
Medroxyprogesterone Acetate	178	0	1	-1	-1	-1	0	0	0	0	0.7392
Epirubicin	150	0	1	0	0	0	0	0	0	0	0.7392
Flutamide	89	0	1	-1	-1	-1	0	0	0	0	0.7392
Irbesartan	79	0	1	-1	-1	-1	0	0	0	0	0.7392
Ampicillin	54	0	1	0	0	0	0	0	0	0	0.7392
Betamethasone	69	0	1	0	0	0	0	0	0	0	0.7392

Mycop henolic Acid	153	0	0	-1	1	1	0	1	0	0	0.7314
Raloxif ene Hydroc hloride	94	0	0	-1	-1	1	0	1	0	0	0.7314
Dacarb azine	250	0	0	1	-1	-1	0	1	0	0	0.7314
Fluoro uracil	51	0	0	-1	1	-1	0	1	0	0	0.7314
Promet hazine	273	0	0	-1	-1	1	0	1	-1	0	0.7314
Efavire nz	143	0	0	-1	-1	1	0	1	0	0	0.7314
Mifepri stone	58	0	0	-1	-1	1	0	1	0	0	0.7314
Pomali domide	234	0	0	-1	1	-1	0	1	0	0	0.7314
Rosigli tazone	122	0	0	-1	-1	1	0	1	0	0	0.7314
Olmesa rtan Medox omil	42	0	0	1	-1	-1	0	1	0	0	0.7314
Acetaz olamid e	78	0	0	-1	1	-1	0	1	-1	0	0.7314
Zidovu dine	164	0	0	1	-1	-1	0	1	0	0	0.7314
Cilosta zol	294	0	0	-1	1	-1	0	1	0	0	0.7314
Mefena mic Acid	295	0	0	1	-1	-1	0	1	0	0	0.7314
Ospemi fene	113	0	0	-1	-1	1	0	1	0	0	0.7314
Gemcit abine	146	0	0	1	-1	-1	0	1	0	0	0.7314
Terbina fine	111	0	0	1	-1	-1	0	1	0	0	0.7314
Benaze pril	31	0	0	-1	-1	1	0	1	0	0	0.7314
Dipyrid amole	258	0	-1	-1	-1	-1	0	0	0	1	0.665
Indome thacin	141	0	-1	-1	-1	-1	0	0	0	1	0.665
Telmis artan	65	0	-1	-1	-1	-1	0	0	0	1	0.665
Disulfir am	90	0	-1	-1	-1	1	0	1	0	0	0.6198
Cyclop hospha mide	73	0	-1	1	-1	-1	0	1	0	0	0.6198
Triclos an	61	0	-1	1	-1	-1	0	1	0	0	0.6198
Ketoco nazole	133	0	-1	1	-1	-1	0	1	0	0	0.6198
Diethyl stilbest rol	40	0	-1	-1	-1	1	0	1	0	0	0.6198
Albend azole	123	0	-1	1	-1	-1	0	1	0	0	0.6198
Mannit ol	241	0	0	0	0	0	0	0	0	0	0.5
Chloro quine Diphos phate	59	0	0	0	0	0	0	0	0	0	0.5

Isoflurophate	242	0	0	0	0	0	0	0	0	0	0.5
Acetamide	238	0	0	0	0	0	0	0	0	0	0.5
Digoxin	239	0	0	-1	-1	-1	0	0	-1	0	0.5
Allergens	244	0	0	0	0	0	0	0	0	0	0.5
Azathioprine	247	0	0	-1	-1	-1	0	0	0	0	0.5
Pioglitazone	52	0	0	0	0	0	0	0	0	0	0.5
Amphotericin B	243	0	0	-1	-1	-1	0	0	0	0	0.5
Colforsin	204	0	0	0	0	0	0	0	0	0	0.5
Aspartame	237	0	0	0	0	0	0	0	0	0	0.5
Neomycin	235	0	0	0	0	0	0	0	0	0	0.5
Nitroglycerin	213	0	0	0	0	0	0	0	-1	0	0.5
Sunitinib	67	0	0	0	0	0	0	0	0	0	0.5
Active Hexose Correlated Compound	216	0	0	0	0	0	0	0	0	0	0.5
Amlexanox	217	0	0	-1	-1	-1	0	0	0	0	0.5
Atrazine	66	0	0	0	0	0	0	0	0	0	0.5
Olmesartan	64	0	0	-1	-1	-1	0	0	0	0	0.5
Penicillamine	223	0	0	0	0	0	0	0	0	0	0.5
Ammonia	224	0	0	0	0	0	0	0	0	0	0.5
Bisphenol A	63	0	0	0	0	0	0	0	0	0	0.5
Codeine	226	0	0	0	0	0	0	0	0	0	0.5
Epinephrine	227	0	0	-1	-1	-1	0	0	-1	0	0.5
Zoledronic Acid	228	0	0	0	0	0	0	0	0	0	0.5
Fluticasone	229	0	0	0	0	0	0	0	0	0	0.5
Phenformin	230	0	0	0	0	0	0	0	0	0	0.5
Water	231	0	0	0	0	0	0	0	0	0	0.5
Cobalt	232	0	0	0	0	0	0	0	0	0	0.5
Zileuton	233	0	0	-1	-1	-1	0	0	0	0	0.5
Norepinephrine	249	0	0	0	0	0	0	0	-1	0	0.5
Moxifloxacin	260	0	0	0	0	0	0	0	0	0	0.5
Carnitine	251	0	0	0	0	0	0	0	0	0	0.5
Rifaximin	252	0	0	-1	-1	-1	0	0	0	0	0.5
Silver	41	0	0	0	0	0	0	0	0	0	0.5

Sodium Chloride	278	0	0	0	0	0	0	0	0	0	0.5
Vitamin K 3	280	0	0	0	0	0	0	0	0	0	0.5
Ofloxacin	282	0	0	0	0	0	0	0	0	0	0.5
Furan	39	0	0	0	0	0	0	0	0	0	0.5
Gadodiamide	37	0	0	0	0	0	0	0	0	0	0.5
Bleomycin	35	0	0	0	0	0	0	0	0	0	0.5
Alpha-Tocopherol	286	0	0	0	0	0	0	0	0	0	0.5
Fluvastatin	287	0	0	0	0	0	0	0	0	0	0.5
Niacinamide	288	0	0	0	0	0	0	0	0	0	0.5
Formoterol Fumarate	290	0	0	0	0	0	0	0	0	0	0.5
Ursodeoxycholic Acid	291	0	0	0	0	0	0	0	0	0	0.5
Mechlorethamine	292	0	0	0	0	0	0	0	0	0	0.5
Pamidronate	293	0	0	0	0	0	0	0	0	0	0.5
Penicillin V	296	0	0	0	0	0	0	0	0	0	0.5
Ethinyl Estradiol	33	0	0	0	0	0	0	0	0	0	0.5
Methimazole	298	0	0	-1	-1	-1	0	0	0	0	0.5
Gliclazide	276	0	0	-1	-1	-1	0	0	0	0	0.5
Ascorbic Acid	46	0	0	0	0	0	0	0	-1	0	0.5
Vardenafil Dihydrochloride	272	0	0	0	0	0	0	0	0	0	0.5
Vinblastine	261	0	0	0	0	0	0	0	0	0	0.5
Vitamin E	253	0	0	0	0	0	0	0	0	0	0.5
Hesperetin	254	0	0	0	0	0	0	0	0	0	0.5
Zomepirac	255	0	0	0	0	0	0	0	0	0	0.5
Tigecycline	256	0	0	-1	-1	-1	0	0	0	0	0.5
Sesamol	257	0	0	0	0	0	0	0	0	0	0.5
Alendronate	259	0	0	0	0	0	0	0	0	0	0.5
Floxacillin	211	0	0	0	0	0	0	0	0	0	0.5
Apigenin	262	0	0	0	0	0	0	0	0	0	0.5
Phenobarbital	271	0	0	0	0	0	0	0	-1	0	0.5

Ethanol	50	0	0	0	0	0	0	0	0	0	0.5
Taurocholic Acid	265	0	0	0	0	0	0	0	0	0	0.5
Sulfapyridine	266	0	0	-1	-1	-1	0	0	0	0	0.5
Dapsone	49	0	0	-1	-1	-1	0	0	0	0	0.5
Salmeterol Xinafoate	268	0	0	-1	-1	-1	0	0	0	0	0.5
Resveratrol	269	0	0	-1	-1	-1	0	0	0	0	0.5
Arsenic Trioxide	47	0	0	0	0	0	0	0	0	0	0.5
Carmustine	212	0	0	-1	-1	-1	0	0	0	0	0.5
Cimetidine	300	0	0	-1	-1	-1	0	0	0	0	0.5
Cocaine	68	0	0	0	0	0	0	0	0	0	0.5
Ciprofloxacin	151	0	0	-1	-1	-1	0	0	-1	0	0.5
Fulvestrant	139	0	0	-1	-1	-1	0	0	0	0	0.5
Chlorine	140	0	0	0	0	0	0	0	0	0	0.5
Imatinib Mesylate	142	0	0	0	0	0	0	0	0	0	0.5
Rifampin	144	0	0	0	0	0	0	0	0	0	0.5
Menthol	145	0	0	0	0	0	0	0	0	0	0.5
Permethrin	147	0	0	-1	-1	-1	0	0	0	0	0.5
Phenytoin	148	0	0	0	0	0	0	0	0	0	0.5
Perindopril	93	0	0	-1	-1	-1	0	0	0	0	0.5
Trovafloxacin	152	0	0	0	0	0	0	0	0	0	0.5
Succimer	96	0	0	0	0	0	0	0	0	0	0.5
Topotecan	155	0	0	0	0	0	0	0	0	0	0.5
Beclomethasone	88	0	0	-1	-1	-1	0	0	0	0	0.5
Lansoprazole	157	0	0	0	0	0	0	0	-1	0	0.5
Estrogens	158	0	0	0	0	0	0	0	0	0	0.5
Dextroamphetamine	159	0	0	0	0	0	0	0	0	0	0.5
Arsenite	160	0	0	0	0	0	0	0	0	0	0.5
Dronabinol	161	0	0	0	0	0	0	0	0	0	0.5
Pentamidine	87	0	0	0	0	0	0	0	0	0	0.5
Mitomycin	138	0	0	0	0	0	0	0	0	0	0.5
Tetrachlorodib	135	0	0	0	0	0	0	0	0	0	0.5

enzodioxin										
Dextromethorphan	165	0	0	0	0	0	0	0	-1	0	0.5
Dust	121	0	0	0	0	0	0	0	0	0	0.5
Ethambutol	106	0	0	0	0	0	0	0	0	0	0.5
Levonorgestrel	105	0	0	0	0	0	0	0	0	0	0.5
Cerivastatin	104	0	0	0	0	0	0	0	0	0	0.5
Cisplatin	116	0	0	0	0	0	0	0	0	0	0.5
Nelfinavir	117	0	0	0	0	0	0	0	0	0	0.5
Diclofenac	118	0	0	0	0	0	0	0	0	0	0.5
Methadone	119	0	0	0	0	0	0	0	0	0	0.5
Pyridostigmine Bromide	102	0	0	-1	-1	-1	0	0	0	0	0.5
Meloxicam	124	0	0	0	0	0	0	0	0	0	0.5
Rofecoxib	134	0	0	-1	-1	-1	0	0	0	0	0.5
Metoprolol	101	0	0	0	0	0	0	0	-1	0	0.5
Pravastatin	126	0	0	0	0	0	0	0	-1	0	0.5
Vancomycin	99	0	0	0	0	0	0	0	-1	0	0.5
Vincristine	128	0	0	0	0	0	0	0	0	0	0.5
Nicotine	129	0	0	0	0	0	0	0	-1	0	0.5
Methoxychlor	97	0	0	0	0	0	0	0	0	0	0.5
Nitrogen	131	0	0	0	0	0	0	0	0	0	0.5
Dihydrotestosterone	132	0	0	0	0	0	0	0	0	0	0.5
Diphenhydramine	209	0	0	0	0	0	0	0	-1	0	0.5
Valdecoxib	163	0	0	-1	-1	-1	0	0	0	0	0.5
Daunorubicin	166	0	0	-1	-1	-1	0	0	0	0	0.5
Allopurinol	188	0	0	-1	-1	-1	0	0	0	0	0.5
Bisoprolol	190	0	0	0	0	0	0	0	0	0	0.5
Irinotecan	167	0	0	0	0	0	0	0	0	0	0.5
Enalaprilat	193	0	0	0	0	0	0	0	0	0	0.5
Polycyclic Aromatic Hydrocarbons	72	0	0	0	0	0	0	0	0	0	0.5
Tacrine	195	0	0	0	0	0	0	0	0	0	0.5

Fexofenadine	196	0	0	0	0	0	0	0	0	0	0.5
Sodium	197	0	0	0	0	0	0	0	0	0	0.5
Erythromycin	198	0	0	0	0	0	0	0	0	0	0.5
Nsc 23766	199	0	0	0	0	0	0	0	0	0	0.5
Roxithromycin	200	0	0	-1	-1	-1	0	0	0	0	0.5
Streptozocin	71	0	0	0	0	0	0	0	0	0	0.5
Dydrogesterone	202	0	0	-1	-1	-1	0	0	0	0	0.5
Oxaliplatin	70	0	0	-1	-1	-1	0	0	0	0	0.5
Quinapril	109	0	0	0	0	0	0	0	0	0	0.5
Kojic Acid	206	0	0	-1	-1	-1	0	0	0	0	0.5
Amiloride	207	0	0	0	0	0	0	0	0	0	0.5
Terfenadine	208	0	0	-1	-1	-1	0	0	0	0	0.5
Silicon Dioxide	74	0	0	0	0	0	0	0	0	0	0.5
Tetracycline	192	0	0	0	0	0	0	0	0	0	0.5
Isoniazid	187	0	0	-1	-1	-1	0	0	0	0	0.5
Dasatinib	77	0	0	-1	-1	-1	0	0	0	0	0.5
Indinavir	168	0	0	0	0	0	0	0	0	0	0.5
Sulfasalazine	169	0	0	-1	-1	-1	0	0	0	0	0.5
Testosterone	86	0	0	0	0	0	0	0	0	0	0.5
Morphine	171	0	0	0	0	0	0	0	-1	0	0.5
Chenodeoxycholic Acid	85	0	0	0	0	0	0	0	0	0	0.5
Apomorphine	174	0	0	-1	-1	-1	0	0	0	0	0.5
Polymyxin B	186	0	0	0	0	0	0	0	0	0	0.5
Isoproterenol	177	0	0	0	0	0	0	0	0	0	0.5
Vinorelbine	176	0	0	0	0	0	0	0	0	0	0.5
Furosemide	179	0	0	-1	-1	-1	0	0	-1	0	0.5
Calcitriol	180	0	0	-1	-1	-1	0	0	-1	0	0.5
Amitriptyline	181	0	0	0	0	0	0	0	-1	0	0.5
Erlotinib Hydrochloride	76	0	0	0	0	0	0	0	0	0	0.5
Oxytetracycline	183	0	0	-1	-1	-1	0	0	0	0	0.5
Crack Cocaine	184	0	0	0	0	0	0	0	0	0	0.5

Thioctic Acid	185	0	0	-1	-1	-1	0	0	0	0	0.5
Warfarin	289	0	-1	0	0	0	0	0	-1	0	0.3744
Mesalamine	221	0	-1	0	0	0	0	0	0	0	0.3744
Lidocaine	220	0	-1	-1	-1	-1	0	0	-1	0	0.3744
Genistein	215	0	-1	-1	-1	-1	0	0	0	0	0.3744
Enalapril	279	0	-1	0	0	0	0	0	0	0	0.3744
Bortezomib	107	0	-1	-1	-1	-1	0	0	0	0	0.3744
Clofibrate	108	0	-1	-1	-1	-1	0	0	0	0	0.3744

Supplementary Table S4. Full list of drug combinations from the top drugs.

Drug A	Drug B	# COVID-19 genes that Drug A hits	# COVID-19 genes that Drug B hits	# COVID-19 genes that either Drug A or B hit	# COVID-19 genes that both Drug A and Drug B hit	type
ETOPOSIDE	SIROLIMUS	2	22	24	0	complementary
MEFLOQUINE	SIROLIMUS	1	22	23	0	complementary
LOSARTAN	RIBAVIRIN	12	6	18	0	complementary
ACETAMINOPHEN	CHLOROQUINE	3	11	14	0	complementary
ETOPOSIDE	LOSARTAN	2	12	14	0	complementary
HYDROXYCHLOROQUINE	MELATONIN	4	10	14	0	complementary
ACETAMINOPHEN	MELATONIN	3	10	13	0	complementary
ATORVASTATIN	CHLOROQUINE	2	11	13	0	complementary
CHLOROQUINE	LOPINAVIR	11	2	13	0	complementary
LOSARTAN	MEFLOQUINE	12	1	13	0	complementary
IVERMECTIN	RIBAVIRIN	6	6	12	0	complementary
ETOPOSIDE	MELATONIN	2	10	12	0	complementary
AZITHROMYCIN	MELATONIN	2	10	12	0	complementary
ATORVASTATIN	MELATONIN	2	10	12	0	complementary
CHLOROQUINE	HEPARIN	11	1	12	0	complementary
HEPARIN	MELATONIN	1	10	11	0	complementary
MEFLOQUINE	MELATONIN	1	10	11	0	complementary
ASPIRIN	RIBAVIRIN	4	6	10	0	complementary
HYDROXYCHLOROQUINE	RIBAVIRIN	4	6	10	0	complementary
HYDROXYCHLOROQUINE	IVERMECTIN	4	6	10	0	complementary
NIFEDIPINE	RIBAVIRIN	4	6	10	0	complementary
ACETAMINOPHEN	RIBAVIRIN	3	6	9	0	complementary
HYDROXYCHLOROQUINE	NIFEDIPINE	4	4	8	0	complementary
LOPINAVIR	RIBAVIRIN	2	6	8	0	complementary
HYDROXYCHLOROQUINE	TENOFOVIR	4	4	8	0	complementary
ETOPOSIDE	RIBAVIRIN	2	6	8	0	complementary
IVERMECTIN	LOPINAVIR	6	2	8	0	complementary
AZITHROMYCIN	RIBAVIRIN	2	6	8	0	complementary
ATORVASTATIN	IVERMECTIN	2	6	8	0	complementary
ATORVASTATIN	RIBAVIRIN	2	6	8	0	complementary
ASPIRIN	HYDROXYCHLOROQUINE	4	4	8	0	complementary
CYCLOSPORINE	RIBAVIRIN	2	6	8	0	complementary
MEFLOQUINE	RIBAVIRIN	1	6	7	0	complementary
HEPARIN	IVERMECTIN	1	6	7	0	complementary

HEPARIN	RIBAVIRIN	1	6	7	0	complementary
ACETAMINOPHEN	TENOFOVIR	3	4	7	0	complementary
ACETAMINOPHEN	HYDROXYCHLOROQUINE	3	4	7	0	complementary
ACETAMINOPHEN	NIFEDIPINE	3	4	7	0	complementary
CYCLOSPORINE	TENOFOVIR	2	4	6	0	complementary
LOPINAVIR	TENOFOVIR	2	4	6	0	complementary
HYDROXYCHLOROQUINE	LOPINAVIR	4	2	6	0	complementary
ETOPOSIDE	HYDROXYCHLOROQUINE	2	4	6	0	complementary
ETOPOSIDE	NIFEDIPINE	2	4	6	0	complementary
CYCLOSPORINE	HYDROXYCHLOROQUINE	2	4	6	0	complementary
AZITHROMYCIN	HYDROXYCHLOROQUINE	2	4	6	0	complementary
ETOPOSIDE	TENOFOVIR	2	4	6	0	complementary
ATORVASTATIN	NIFEDIPINE	2	4	6	0	complementary
ATORVASTATIN	HYDROXYCHLOROQUINE	2	4	6	0	complementary
ATORVASTATIN	TENOFOVIR	2	4	6	0	complementary
ASPIRIN	ETOPOSIDE	4	2	6	0	complementary
AZITHROMYCIN	TENOFOVIR	2	4	6	0	complementary
ASPIRIN	CYCLOSPORINE	4	2	6	0	complementary
ASPIRIN	AZITHROMYCIN	4	2	6	0	complementary
ASPIRIN	ATORVASTATIN	4	2	6	0	complementary
HEPARIN	NIFEDIPINE	1	4	5	0	complementary
MEFLOQUINE	TENOFOVIR	1	4	5	0	complementary
MEFLOQUINE	NIFEDIPINE	1	4	5	0	complementary
HYDROXYCHLOROQUINE	MEFLOQUINE	4	1	5	0	complementary
HEPARIN	TENOFOVIR	1	4	5	0	complementary
ASPIRIN	HEPARIN	4	1	5	0	complementary
ACETAMINOPHEN	ATORVASTATIN	3	2	5	0	complementary
ACETAMINOPHEN	ETOPOSIDE	3	2	5	0	complementary
ACETAMINOPHEN	CYCLOSPORINE	3	2	5	0	complementary
ACETAMINOPHEN	AZITHROMYCIN	3	2	5	0	complementary
ACETAMINOPHEN	LOPINAVIR	3	2	5	0	complementary
ASPIRIN	MEFLOQUINE	4	1	5	0	complementary
ETOPOSIDE	LOPINAVIR	2	2	4	0	complementary
CYCLOSPORINE	LOPINAVIR	2	2	4	0	complementary
AZITHROMYCIN	LOPINAVIR	2	2	4	0	complementary
ATORVASTATIN	ETOPOSIDE	2	2	4	0	complementary

ATORVASTATIN	AZITHROMYCIN	2	2	4	0	complementary
ACETAMINOPHEN	HEPARIN	3	1	4	0	complementary
ACETAMINOPHEN	MEFLOQUINE	3	1	4	0	complementary
ATORVASTATIN	CYCLOSPORINE	2	2	4	0	complementary
ATORVASTATIN	MEFLOQUINE	2	1	3	0	complementary
ATORVASTATIN	HEPARIN	2	1	3	0	complementary
AZITHROMYCIN	HEPARIN	2	1	3	0	complementary
CYCLOSPORINE	HEPARIN	2	1	3	0	complementary
ETOPOSIDE	HEPARIN	2	1	3	0	complementary
HEPARIN	LOPINAVIR	1	2	3	0	complementary
LOPINAVIR	MEFLOQUINE	2	1	3	0	complementary
HEPARIN	MEFLOQUINE	1	1	2	0	complementary
