# Supplementary material for: Drug Repurposing for COVID-19 using Graph Neural Network with Genetic, Mechanistic, and Epidemiological Validation
Source: Res Sq. 2020 Dec 11:rs.3.rs-114758. Preprint. [Version 1] doi: 10.21203/rs.3.rs-114758/v1 (PMC7743080; doi:10.21203/rs.3.rs-114758/v1)

Supplementary Figure S1. The SARS-CoV-2 Knowledge Graph

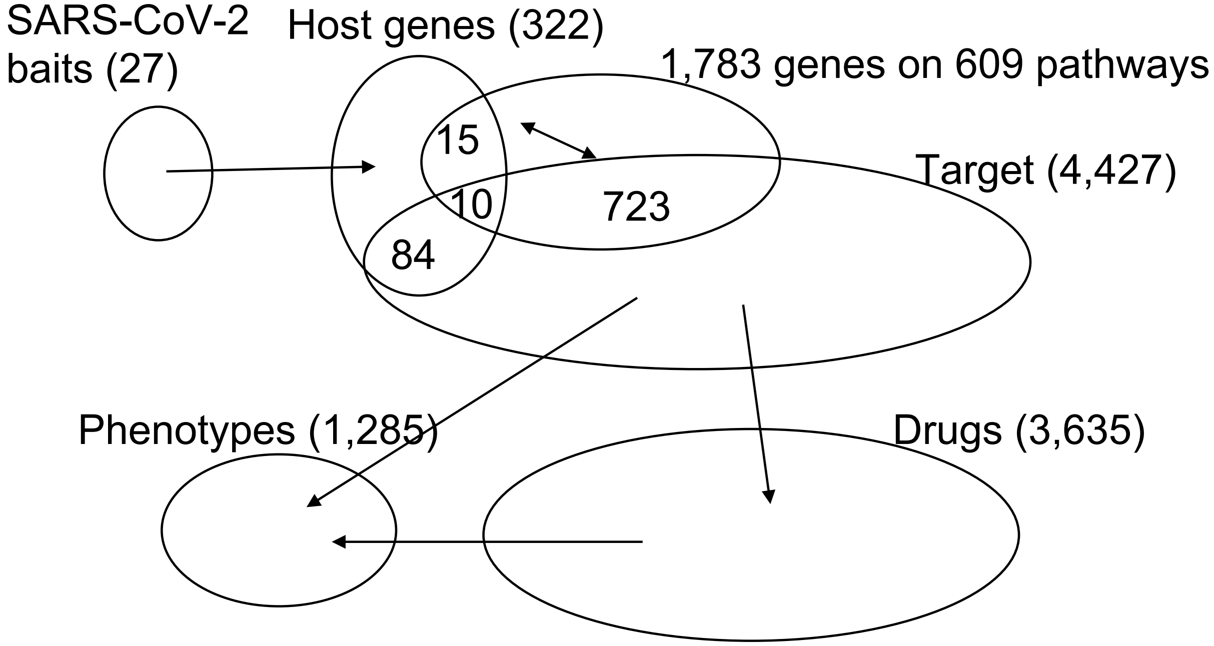

Supplementary Figure S2. Interactive t-sne plot

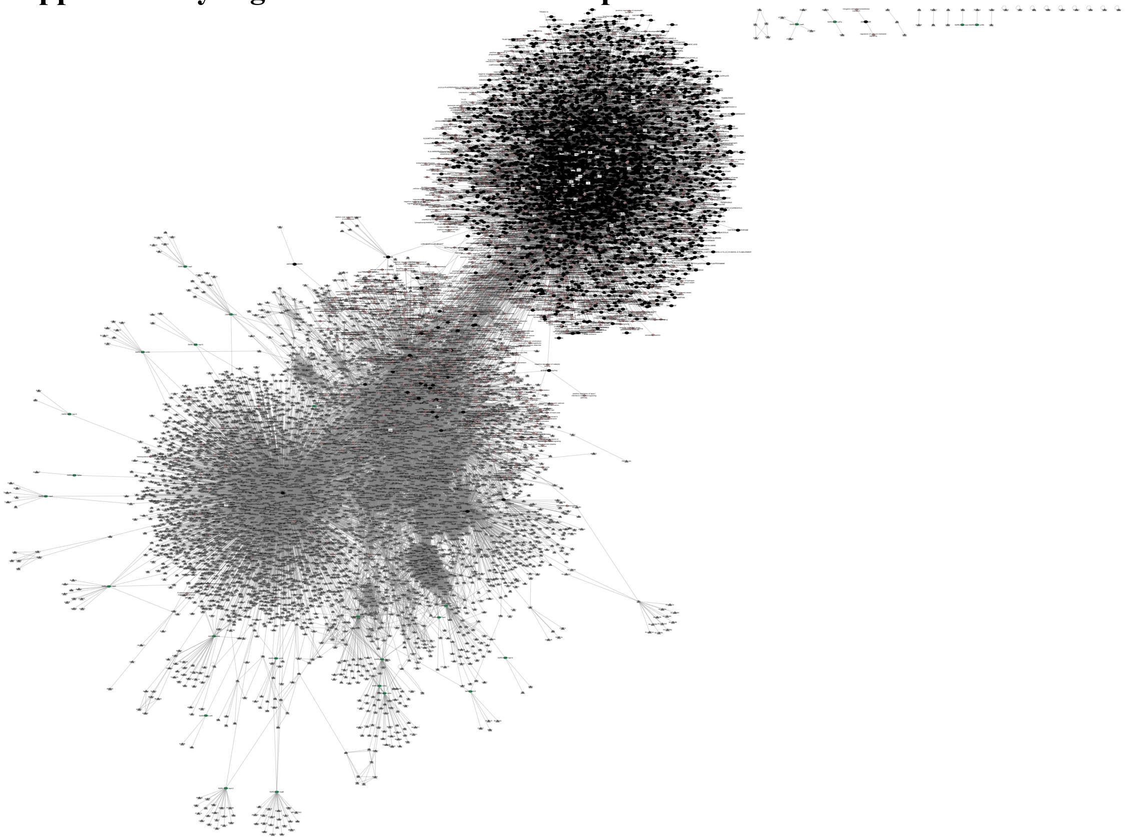

Supplementary Figure S3. External validation (a) Accuracy was measured in the intersection of candidate drugs and external validation sources. (b) Cohort selection and propensity score matching in EHRs.

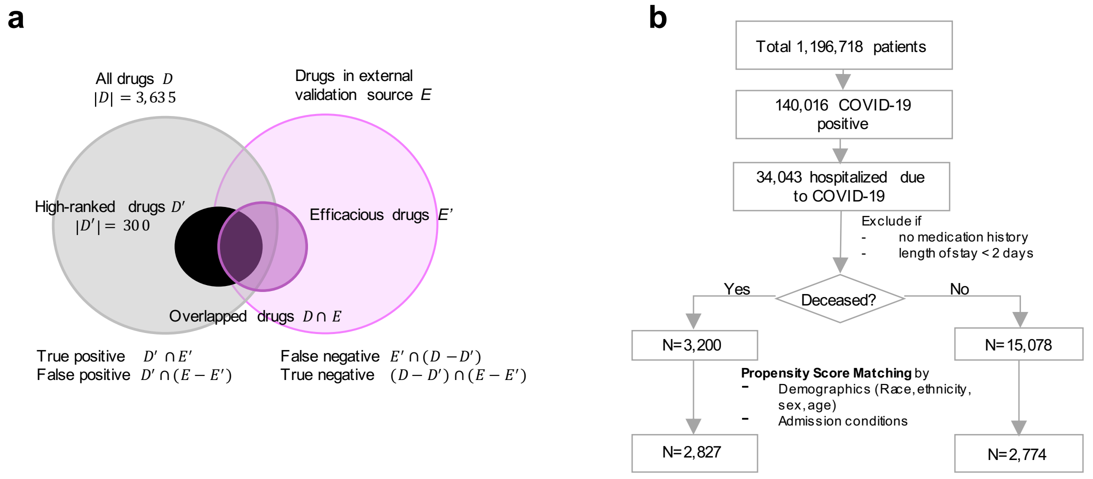

Supplement: Supplement — ary Figure S1. The SARS-CoV-2 Knowledge Graph Supplementary Figure S2. Interactive t-sne plot Supplementary Figure S3. External validation (a) Accuracy was measured in the intersection of candidate drugs and external validation sources. (b) Cohort selection and propensity score matching in EHRs. [file bf6a3eddf4ff2141bf83bef6.pdf]
