# Supplementary material for: Drug Repurposing for COVID-19 using Graph Neural Network with Genetic, Mechanistic, and Epidemiological Validation
Source: Res Sq. 2020 Dec 11:rs.3.rs-114758. Preprint. [Version 1] doi: 10.21203/rs.3.rs-114758/v1 (PMC7743080; doi:10.21203/rs.3.rs-114758/v1)
Supplement: Supplement — ary Note S1 Transfer learning Supplementary Note S2 Calculating the treatment effect Supplementary Note S3 Re-ordering the validated drugs Supplementary Note S4 Genetic validation using gene set enrichment analysis [file a33ee444486abd69101333d4.pdf]

Supplementary Notes

Supplementary Note S1 *Transfer learning*

Drug Repurposing Knowledge Graph (DRKG) comprises 97,238 nodes (from 13 node types including gene, molecular function, pathway, disease, symptom, anatomy, cellular component, compounds, side effect, ATC, and pharmacologic class) and 5,874,261 interactions (from 107 edge types from the 17 entity types). These nodes and interactions are collected from various databases including DrugBank, HetioNet, GNBR, STRING, IntAct, and DGLdb [1]. We utilized the DRKG via transferring DRKG pre-trained node embedding (that is trained with a translation embedding model) [2]. The pre-trained node embedding was used as an initial node embedding in our SARS-CoV-2 knowledge graph, and we fine-tuned the embedding toward the selected SARS-CoV-2 interactions by updating them via the iterative message passing and aggregation. Our SARS-CoV-2 knowledge graph partly overlaps with the DRKG; the number of overlap was 1,138 drugs (out of 3,635), 5,666 genes (out of 5,677), and 970 phenotypes (out of 1,285). The node embedding of the non-overlapped nodes was set as zero vectors initially and updated along with message propagation.

Supplementary Note S2 *Calculating the treatment effect*

We had two steps for balancing the confounders: i) initial case and control matching using propensity score (PSM) and ii) weighting treatment effect using the propensity score. PSM is a principled approach to align patients based on the confounders (e.g., demographics and admission severity) [3]. The propensity score is defined as $\pi(X_i) = f(T_i = 1|X_i)$, where $X_i$is the patient i's covariates; $T_i$is a binary indicator on whether a patient$i$ receives treatment; and $f$is a function to predict the probability of the patient $i$receiving the treatment ($T_i = 1$). In this way, we can represent the likelihood of receiving the treatment at a population level. Calculating the treatment effect is to measure the causal effect of treatments (i.e., candidate drugs) as balancing the confounders between the treated and their counterfactuals based on the likelihood of receiving the treatment. The average treatment effect (ATE) compares the average outcome (deceased$Y_i = 0$ or recovered $Y_i = 1$) in the presence or absence of the treatment$ATE = E(Y_i = 1) - E(Y_i = 0)$. Using the inverse propensity score weighting method [4], ATE can be written as $\widehat{ATE} = \frac{1}{n}\sum_{i=1}^{n} [\frac{T_i Y_i}{\pi(X_i)} - \frac{(1-T_i)Y_i}{1-\pi(X_i)}]$, which intuitively suppresses the impact of highly predictable records (e.g., $\pi(X_i) \sim 1$and$T_i = 1$, $\pi(X_i) \sim 0$and $T_i = 0$) while giving high weight to the rest (i.e., much random in terms of all observed confounders). The average treatment effect on the treated (ATT) is similarly defined as ATE but focused on the among the treated $ATT = E(Y_i = 1|T_i = 1) - E(Y_i = 0|T_i = 1)$that tests the mean differences between those treated vs. their counterfactuals. We can calculate ATT similarly by limiting the sample to the treated patients. ATT is often more realistic than ATE because not every patient has a chance to be treated.

Supplementary Note S3 *Re-ordering the validated drugs*

We present the final validated drugs based on the existence of supportive external evidence. We have genetic (GSEA score), mechanistic (four in-vitro drug screening results), and population-based (EHRs) evidence. These validation sources only covered a part of the drug candidates and sometimes conflict with each other. Integrating the different aspects of evidence to identify high-confidence drugs requires a careful aggregation method. We preferred the population-based evidence over the in-vitro or genetic evidence. Our approach to aggregate the different evidence into a confidence score or ranking is to use data programming, which is implemented in *Snorkel* [5]. Data programming is to aggregate weak labels into a single confidence score by inferring causal dependency among the weak labels. Our rules to define the weak labels are as follows:

- Rule1: If a drug is under trial; then the drug is positive, otherwise unknown,
- Rule2: If a drug was effective in EHRs; then the drug is positive, otherwise unknown,
- Rule3: If a drug is effective in any of four in-vitro experiments; then the drug is positive, otherwise unknown
- Rule3: If a drug is effective in GSEA; then the drug is positive, otherwise unknown
- Rule4: If a drug was not effective in EHRs nor efficacious in all in-vitro screening; then the drug is negative, otherwise unknown

Supplementary Note S4 *Genetic validation using gene set enrichment analysis*

For the genetic validation, we evaluated the drugs by calculating GSEA scores between gene expression profiles of SARS-CoV-2-infected host cells and the gene signature of the drugs. The SARS-CoV-2 genetic profile was three samples from SARS-CoV-2 infected primary human airway epithelial cell lines[6] and three mock-infected (PBS) cell lines (GSE153970). *Deseq2* was used to detect the differentially expressed genes (DEGs) by adjusted p-value less than 0.01 [7]. The up-regulated and down-regulated genes from DEGs were considered as an up-regulated SARS-CoV-2 signature and down-regulated SARS-CoV-2 signature. The drug's genetic profiles were obtained from the drug-induced gene expression in cMAP (GSE92742 and GSE70138)(Subramanian et al. 2017). The whole drugs' gene probe set was ordered from the highest up-regulated genes to the lowest down-regulated genes.

The enrichment score (ES) was calculated to reflect the correlation between the SARS-CoV-2 signature and the drug's gene expression by connectivity map algorithms [8]. The hypothesis was that if the drug's gene expression is opposite with the disease up-regulated or down-regulated signature, the drug tends to treat disease [9]. ES is calculated as follows [10]:

$$ES = (ES_{up} - ES_{down})/2 \; if \; sgn(ES_{up}) \neq sgn(ES_{down}); \; else \; 0.$$

ES_{up} is the enrichment score for SARS-CoV-2 up-regulated signature; ES_{down} is the enrichment score for SARS-CoV-2 down-regulated signature. If ES_{up} and ES_{down} have the same algebraic sign, the value of final ES is set to 0. Otherwise, ES is the difference between ES_{up} and ES_{down}. ES_{down} and ES_{down} was calculated based on the weighted Kolmogorov-Smirnov enrichment statistic (ES). In order to obtain p-value, permutation tests were done by randomly generating the same number of genes as upregulated gene set and downregulated gene set separately and thus we can get the null distribution of random ES. We identified a significant genetic association between

the drug and the disease by setting a threshold as ES < 0 and p-value <0.05, which means a drug has opposite effects for both up-regulated SARS-CoV-2 (ES_{up}< 0) and down-regulated SARS-CoV-2 set (ES_{down}> 0).

Reference

1. gnn4dr. gnn4dr/DRKG. *GitHub* https://github.com/gnn4dr/DRKG.
2. Antoine Bordes, Nicolas Usunier, Alberto Garcia-Duran, Jason Weston, Oksana Yakhnenko. Translating Embeddings for Modeling Multi-relational Data. in *Advances in Neural Information Processing Systems 26 (NIPS 2013)*.
3. Patorno, E., Grotta, A., Bellocco, R. & Schneeweiss, S. Propensity score methodology for confounding control in health care utilization databases. *Epidemiology, Biostatistics and Public Health* **10**, (2013).
4. Bauer, P. C. 4.15 ATE: Average Treatment Effect. https://bookdown.org/paul/applied-causal-analysis/ate.html.
5. Ratner, A., De Sa, C., Wu, S., Selsam, D. & Ré, C. Data Programming: Creating Large Training Sets, Quickly. *Adv. Neural Inf. Process. Syst.* **29**, 3567–3575 (2016).
6. Vanderheiden, A. *et al.* Type I and Type III IFN Restrict SARS-CoV-2 Infection of Human Airway Epithelial Cultures. *J. Virol.* (2020) doi:10.1128/JVI.00985-20.
7. Love, M. I., Huber, W. & Anders, S. Moderated estimation of fold change and dispersion for RNA-seq data with DESeq2. *Genome Biol.* **15**, 1–21 (2014).
8. Subramanian, A. *et al.* A Next Generation Connectivity Map: L1000 Platform and the First 1,000,000 Profiles. *Cell* **171**, 1437–1452.e17 (2017).
9. Sirota, M. *et al.* Discovery and preclinical validation of drug indications using compendia of public gene expression data. *Sci. Transl. Med.* **3**, 96ra77 (2011).
10. Lamb, J. The Connectivity Map: Using Gene-Expression Signatures to Connect Small Molecules, Genes, and Disease. *Science* vol. 313 1929–1935 (2006).
