# Supplementary material for: Drug Repurposing for COVID-19 using Graph Neural Network with Genetic, Mechanistic, and Epidemiological Validation
Source: Res Sq. 2020 Dec 11:rs.3.rs-114758. Preprint. [Version 1] doi: 10.21203/rs.3.rs-114758/v1 (PMC7743080; doi:10.21203/rs.3.rs-114758/v1)
Supplement: Supplement [file d493e8e643e9a62f141949e1.pdf]

Drug Repurposing for COVID-19 using Graph Neural Network with Genetic, Mechanistic, and Epidemiological Validation

Authors: Kanglin Hsieh, Ph.D.,[1] Yinyin Wang, M.S.,[2] Luyao Chen, M.S.,[1] Zhongming Zhao, Ph.D.,[3] Sean Savitz, M.D.,[4] Xiaoqian Jiang, Ph.D.,[1] Jing Tang, Ph.D.,[2] Yejin Kim, Ph.D.[1*]

Supplementary Information Index

Supplementary Notes 1: Transfer learning
Supplementary Notes 2: Calculating the treatment effect
Supplementary Notes 3: Re-ordering the validated drugs
Supplementary Notes 4: Genetic validation using gene set enrichment analysis

Supplementary Table S1: Link prediction accuracy in the SARS-CoV-2 knowledge graph.
Supplementary Table S2: ATT score of 138 drugs that were in EHRs and initial 3,635 drugs.
Supplementary Table S3: Full list of repurposable drugs
Supplementary Table S4: Full list of drug combinations from the top drugs.

Supplementary Figure S1: The SARS-CoV-2 Knowledge Graph
Supplementary Figure S2: Interactive t-sne plot
Supplementary Figure S3: External validation (a) Accuracy was measured in the intersection of candidate drugs and external validation sources. (b) Cohort selection and propensity score matching in EHRs.
